# Tethering of Multi-Vesicular Bodies and the Tonoplast to the Plasma Membrane in Plants

**DOI:** 10.3389/fpls.2019.00636

**Published:** 2019-05-22

**Authors:** Kai Tao, Justin R. Waletich, Hua Wise, Felipe Arredondo, Brett M. Tyler

**Affiliations:** ^1^Molecular and Cellular Biology Program, Oregon State University, Corvallis, OR, United States; ^2^Department of Botany and Plant Pathology, Oregon State University, Corvallis, OR, United States; ^3^Center for Genome Research and Biocomputing, Oregon State University, Corvallis, OR, United States

**Keywords:** BiFC, plasma membrane, tonoplast, multi-vesicular bodies, tethering

## Abstract

Tethering of the plasma membrane (PM) and many organelles to the endoplasmic reticulum (ER) for communication and lipid exchange has been widely reported. However, despite growing interest in multi-vesicular bodies (MVBs) as possible sources of exosomes, tethering of MVBs to the PM has not been reported. Here we show that MVBs and the vacuolar membrane (tonoplast) could be tethered to the PM (PM-MVB/TP tethering) by artificial protein fusions or bimolecular fluorescence complementation (BiFC) complexes that contain a peripheral membrane protein that binds the PM and also a protein that binds MVBs or the tonoplast. PM-binding proteins capable of participating in PM-MVB/TP tethering included StRem1.3, BIK1, PBS1, CPK21, and the PtdIns(4)-binding proteins FAPP1 and Osh2. MVB/TP-binding proteins capable of participating in tethering included ARA6, ARA7, RHA1, RABG3f, and the PtdIns(3)P-binding proteins Vam7p and Hrs-2xFYVE. BiFC complexes or protein fusions capable of producing PM-MVB/TP tethering were visualized as large well-defined patches of fluorescence on the PM that could displace PM proteins such as AtFlotillin1 and also could displace cytoplasmic proteins such as soluble GFP. Furthermore, we identified paralogous ubiquitin E3 ligase proteins, SAUL1 (AtPUB44), and AtPUB43 that could produce PM-MVB/TP tethering. SAUL1 and AtPUB43 could produce tethering in uninfected tissue when paired with MVB-binding proteins or when their E3 ligase domain was deleted. When *Nicotiana benthamiana* leaf tissue was infected with *Phytophthora capsici*, full length SAUL1 and AtPUB43 localized in membrane patches consistent with PM-MVB/TP tethering. Our findings define new tools for studying PM-MVB/TP tethering and its possible role in plant defense.

**Significance Statement:**

Although not previously observed, the tethering of multi-vesicular bodies to the plasma membrane is of interest due to the potential role of this process in producing exosomes in plants. Here we describe tools for observing and manipulating the tethering of multi-vesicular bodies and the tonoplast to the plant plasma membrane, and describe two plant proteins that may naturally regulate this process during infection.

## Introduction

In eukaryotic cells, the endosomal system is composed of diverse highly dynamic vesicular organelles performing the functions of cargo storage, sorting, and delivery to specific destinations (Mellman, 1996; Bonifacino and Traub, 2003). Based on their different functions, these vesicular organelles have been generally classified in animal cells as early endosomes (EE), recycling endosomes (REs), and late endosomes (LEs) (also known as multivesicular bodies; MVBs) (Singh et al., 2014; Hu et al., 2015). Heterotypic fusion between endocytic vesicles and the EE is regulated by a small GTPase referred to as Rab5, which also promotes homotypic fusion between EEs (Jovic et al., 2010; Zeigerer et al., 2012). EEs function as the major sorting hub for membrane and soluble cargos. Most membrane cargos are sorted for recycling back to the PM via recycling endosomes (REs) (Ang et al., 2004; van IJzendoorn, 2006; Hsu and Prekeris, 2010). On the other hand, cargos destined for degradation in lysosomes are targeted to regions of the EEs destined to mature into LEs (Huotari and Helenius, 2011). The hallmark of the maturation of EEs to LEs is loss of Rab5 from endosomal membrane with the concomitant acquisition of Rab7 (Poteryaev et al., 2010). LEs move unidirectionally toward, and then fuse with, the lysosomes which contain a variety of hydrolytic enzymes for the turnover of the endocytic cargos (Xu and Ren, 2015).

The endosomal system in plant cells shares major similarities with mammalian systems. However, it has been suggested that plant cells lack distinct EEs and instead that the TGN takes on the function of EEs in receiving endocytic cargos (Dettmer et al., 2006; Chow et al., 2008; Viotti et al., 2010). Furthermore, it was reported that in plants, subdomains of the TGN could either function as REs, or could gradually mature into MVBs which correspond to LEs in mammalian cells (Scheuring et al., 2011; Singh et al., 2014). MVBs in plants have been generally identified as an intermediate hub, where endocytic cargo can either undergo retrograde trafficking to the TGN or be targeted to the lytic compartments for degradation (Cui et al., 2016). Moreover, plant MVBs also appear to function in the sorting of biosynthetic endosomes destined for the vacuole, and therefore are often termed prevacuolar compartments (PVCs) (Tse et al., 2004; Shen et al., 2011; Contento and Bassham, 2012).

A further major difference in plant cells is that lysosomes are replaced by vacuoles as the end-point of the endocytic pathway for degradation. In many but not all plant cells, vacuoles occupy more than 90% of the total cell volume (Festa et al., 2016). The vacuole also carries out important functions such as maintaining cellular homeostasis (e.g., pH, redox, osmolarity) (Zhu, 2001; Hurth et al., 2005; Andreev, 2012), contributing to the immune response against pathogens via programmed cell death or discharge of anti-microbial vacuolar contents (Hatsugai and Hara-Nishimura, 2010; Hatsugai et al., 2015), and regulating cell volume in support of the structural integrity of plants (Reisen et al., 2005). The vacuolar membrane, also called the tonoplast, is the location for the fusion of TGN-derived MVBs with vacuoles (Scheuring et al., 2011).

In the endocytic pathway, the lipid composition of the endosomal membranes is a major determinant of the identity, function and differentiation of the various vesicular components of the system. The lipids not only influence the biophysical properties of the membrane bilayers, but they also recruit specific endosomal effector proteins that mediate vesicle targeting, sorting, fusion, and docking. In animal endosomal systems, phosphatidylinositol 3-phosphate (PtdIns(3)P) is a defining characteristic of the EEs (Di Paolo and De Camilli, 2006). GTP-bound Rab5 on the endosomal membrane can interact with the effector protein phosphoinositide 3-kinase (PI3K) resulting in local synthesis of PtdIns(3)P (Murray et al., 2002; Jovic et al., 2010). The presence of PtdIns(3)P establishes the identity of EEs by recruiting a variety of effector proteins that contain PtdIns(3)P-binding modules such as the FYVE domain of early endosomal antigen-1 (EEA1) of human cells (Gaullier et al., 1998), the Phox (PX) domain of the Qc-SNARE (soluble NSF attachment protein receptor), and Vam7p of yeast cells (Sato et al., 2001).

In plant cells, although the TGN appears to functionally replace the role of EEs, it is usually devoid of PtdIns(3)P (Paez Valencia et al., 2016). Instead, PtdIns(3)P is highly enriched on the MVBs and also occurs on the tonoplast, where it has been visualized by using fluorescently tagged PtdIns(3)P-specific biosensors (Vermeer et al., 2006; Simon et al., 2014). These results are in accordance with the finding that a pair of functionally redundant canonical Rab5-type GTPases, RABF2b/ARA7 (Bottanelli et al., 2012) and RABF2a/RHA1 (Sohn et al., 2003), together with a plant-specific Rab5-like GTPase RABF1/ARA6 (Ebine et al., 2011) are located on MVBs. In addition, a Rab7-type GTPase, RABG3f, is localized on MVBs and the tonoplast, mediating vesicular trafficking to the vacuoles (Nahm et al., 2003; Cui et al., 2014). Thus, in plants, PtdIns(3)P is characteristic of the system of vesicles and membranes that functionally replaces the LEs of animal cells.

A set of membrane-bound vesicles that have gained renewed attention recently, especially in the context of plant-microbe interactions, are vesicles referred to as exosomes that are released into the intercellular environment. Exosomes, which are one type of extracellular vesicles, have been described from fungi, plants, and animals, including cancer cells (Samuel et al., 2015; Schorey et al., 2015; Maas et al., 2017). Exosomes appear to originate from a variety of sources, particularly MVBs, and appear to be involved in transport of a variety of chemicals and proteins into the extracellular space. They also have the potential to deliver their contents into adjacent cells, including those of invading microbes. One path for the release of exosomes appears to involve membrane fusion between MVBs and the PM (Théry et al., 2002; Hanson and Cashikar, 2012; Colombo et al., 2014). In plants, exosomes are also appear to be produced from MVBs, and in particular are found to be increased in abundance in response to biotic or abiotic stress (An et al., 2007; Samuel et al., 2015; Rutter and Innes, 2017). However, little is known about the machinery of this process.

In the process of mapping the locations of phosphatidylinositol-3-phospate (PtdIns(3)P) relative to a series of sub-cellular marker proteins inside plant cells, we observed that protein fusions, or bimolecular fluorescence complementation (BiFC) complexes, that contain one domain that binds the PM and one domain that binds MVBs or the tonoplast, could produce structures consistent with the tethering of those organelles to the PM. Constructs derived from E3 ubiquitin ligases SAUL1 and AtPUB43 could produce similar tethering-like structures in healthy *Nicotiana benthamiana* leaf tissue, and the full length proteins were associated with tethering-like structures that formed during infection of *N. benthamiana* by the oomycete *P. capsici*.

## Results

### Biosensors for PtdIns(3)P, Endosomes, MVBs, the Tonoplast, and the PM

Since the initial motivation for this study was to map the locations of PtdIns(3)P, we firstly created two YFP-containing biosensors that could enable the visualization of PtdIns(3)P. One biosensor, VAM7-PX-YFP, contained a phox homology (PX) domain from the *Saccharomyces cerevisiae* Qc-SNARE protein, VAM7p. The other biosensor comprised a tandem repeat of the FYVE domain from the rat hepatocyte growth factor-regulated tyrosine kinase substrate, namely Hrs-2xFYVE-YFP. Both proteins have been well-characterized as specifically recognizing PtdIns(3)P *in vitro* and in yeast, animal and plant cells (Komada and Kitamura, 1995; Cheever et al., 2001; Lee et al., 2006; Vermeer et al., 2006; He et al., 2011). We used *Agrobacterium tumefaciens*-mediated transient transformation to ectopically express the VAM7-PX-YFP or Hrs-2xFYVE-YFP fusion proteins in *N. benthamiana* leaf cortical cells. Then the leaf tissue was examined by confocal fluorescence microscopy. To obtain a comprehensive view of the plasma membrane, Z-axis scanning imaging was utilized to build 3D visualizations via the maximal intensity projection. As shown in Supplementary Figure S1, the fluorescence produced by VAM7-PX-YFP and Hrs-2xFYVE-YFP was observed on motile vesicular organelles, and on the tonoplast. These locations were verified by co-localization analyses using the well-characterized endosome markers ARA6 and RABG3f and the tonoplast marker AtTPK1 (Supplementary Figure S2). This observation aligns with previous studies in tobacco BY-2 cells (Vermeer et al., 2006) and *Arabidopsis* root cells (Simon et al., 2014). Additionally, to confirm that PtdIns(3)P-binding was required for subcellular localizations of the biosensors, we designed mutations in the PtdIns(3)P-binding sites of the biosensor proteins through site-directed mutagenesis as previously described (Kutateladze and Overduin, 2001; Lee et al., 2006; Pankiv et al., 2010). Both mutant biosensors, VAM7-PX^*^ and Hrs-2xFYVE^*^, completely lost targeting to any membrane, including the MVBs and tonoplast; instead they accumulated in the cytoplasm (Supplementary Figure S1). Though both PtdIns(3)P biosensors appeared to predominately target the tonoplast as well as the endosomes, the close apposition of the tonoplast to the PM due to turgor pressure (Reisen et al., 2005) made it difficult to rule out the presence of some level of PtdIns(3)P on the inner leaflet of the PM (Supplementary Figure S2), as visualized by the widely used PM marker protein, the remorin StRem1.3 (Perraki et al., 2012; Jarsch et al., 2014).

### BiFC Complexes Containing Ptdins(3)P Biosensors and StRem1.3 Produce Large Patches of Plasma Membrane Fluorescence

To more unambiguously address whether PtdIns(3)P was located only on the tonoplast and not on the PM, we used the bimolecular fluorescence complementation (BiFC) assay. Normally in this technique, two non-fluorescing fragments of a fluorescent protein are translationally fused with proteins of interest. If the proteins of interest bind to each other, the two non-fluorescing fragments are brought into proximity resulting in the re-assembly of a functional fluorescent protein (Kerppola, 2006). However, actual physical interaction between the proteins of interest is not always required, and it may be sufficient to bring the proteins into near proximity, e.g., by binding to the same membrane domain. Furthermore, if the fluorescent protein used in the BiFC assays matures quickly, and is highly expressed, the two non-fluorescing fragments may associate into a functional protein as a result of random transient contacts, and direct association of the proteins of interest may not be required.

**Figure 1:**
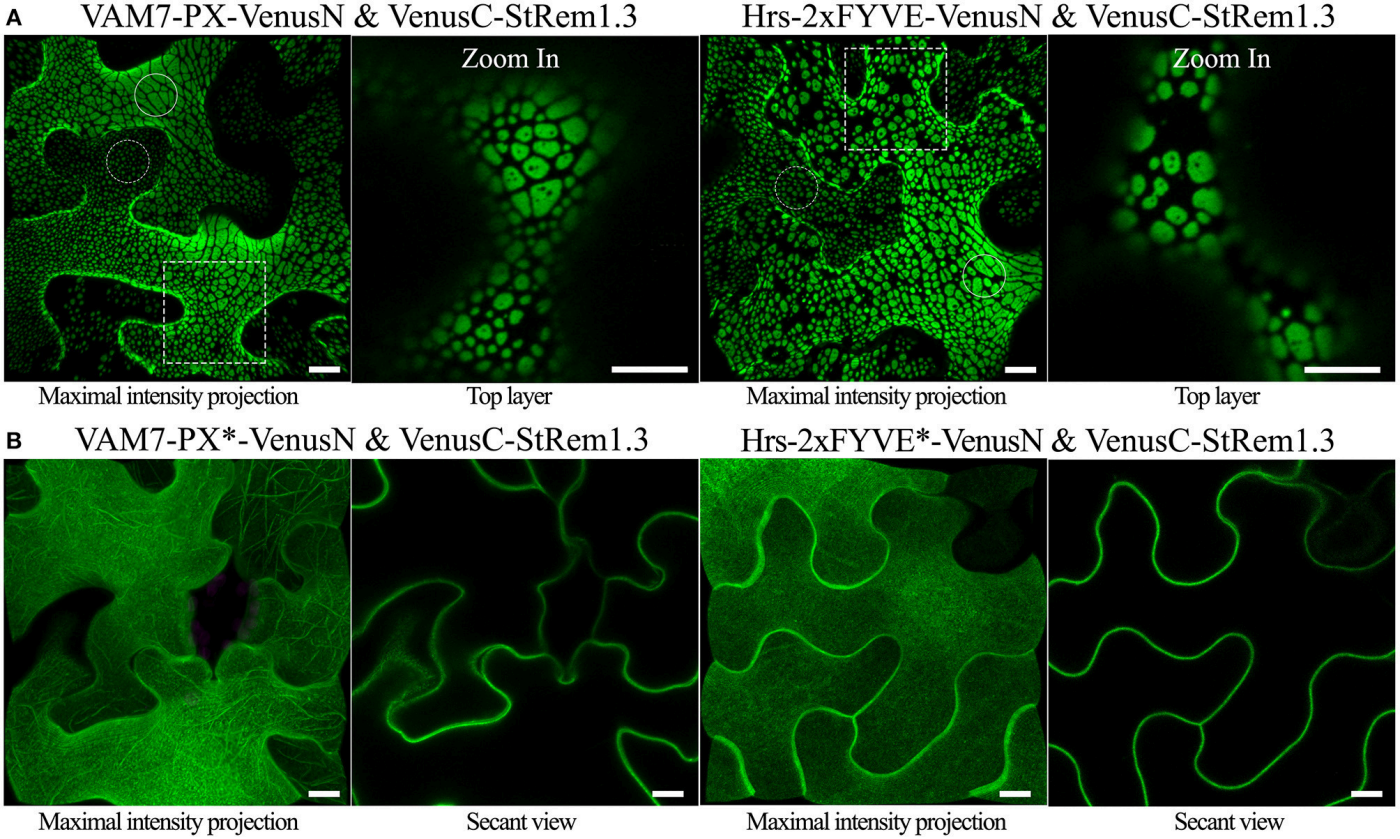
Co-expression of PtdIns(3)P-binding proteins and StRem1.3 produces BiFC complexes distributed in large patches on the PM of *N. benthamiana* leaf cortical cells. **(A)** BiFC fluorescence in cells co-expressing VAM7-PX-VenusN or Hrs-2xFYVE-VenusN with VenusC-StRem1.3. The dotted boxes indicate the regions zoomed in in the top layer projections. **(B)** BiFC fluorescence in cells co-expressing VenusC-StRem1.3 with VenusN fusions to the PtdIns(3)P-non-binding mutants, VAM7-PX^*^, Hrs-2xFYVE^*^. Large membrane patches are highlighted by the solid circles, and small punctae are highlighted with the dotted circles.All scale bars represent 10 μm.

To examine the sub-cellular proximity of PtdIns(3)P and StRem1.3, we fused VAM7-PX to an N-terminal fragment of Venus FP (1–155; VenusN) and StRem1.3 was fused to a C-terminal fragment (156–239; VenusC). We expected that if VAM7-PX-VenusN targeted PM PtdIns(3)P then its co-expression with PM-localized VenusC-StRem1.3 might result in a fluorescent signal from re-assembled Venus, especially if PtdIns(3)P was localized in the same PM microdomains as StRem1.3. In fact, we did observe significant BiFC fluorescent signals from this experiment. Surprisingly however, the BiFC fluorescent signal was distributed into patches of varying sizes across most of the surfaces of the cortical cells (Figure 1A). The sizes ranged from extensive patches down to small punctae (highlighted by solid and dotted circles in Figure 1A). The larger patches typically had a variety of small, round, non-fluorescent inclusions. A similar distribution of BiFC fluorescence was also observed in complexes containing Hrs-2xFYVE (Figure 1A). On the other hand, this pattern was not observed in BiFC complexes containing the mutant biosensors VAM7-PX^*^ and Hrs-2xFYVE^*^; instead those complexes were homogeneously distributed on the plasma membrane (Figure 1B), closely matching the localization of fusions of StRem1.3 with full-length YFP (Supplementary Figure S2). Thus, PtdIns(3)P-binding appeared to be required for formation of the patches. A similar pattern, ranging from large patches down to small punctae, was observed when the constructs were co-expressed in *Arabidopsis* mesophyll protoplasts (Supplementary Figure S3). Furthermore, since fluorescent Venus BiFC complexes were formed by the mutant biosensors as efficiently as the non-mutant ones, we concluded that, under the conditions of our assays, the VenusN and VenusC fragments could spontaneously re-assemble without the need for close association of the fused proteins of interest, as noted by others (Gookin and Assmann, 2014).

We also designed chimeric fusion proteins consisting of a PtdIns(3)P biosensor at the N terminus, full length YFP or tagRFP in the middle, and StRem1.3 at the C terminus, that are structurally equivalent to the BiFC complexes used above. Both the YFP and tagRFP versions of the chimeric fusion proteins produced the characteristic pattern of patches observed above (Supplementary Figure S4). Thus, the formation of the patches was not restricted to BiFC experiments.

The sizes of the membrane patches were highly variable among different cells, or even within the same cells (Figure 1A, and Supplementary Figures S3, S4). To test the hypothesis that the differences in sizes resulted from differences in transcript levels encoding these fluorescent protein fusions, we compared the patterns produced by constructs driven by the strong cauliflower mosaic virus 35S promoter (*CaMV35S*) (Sunilkumar et al., 2002) (used for all experiments described above), with those driven by the native promoter of *Arabidopsis AtRem1.4* (At5g23750.1), the closest *Arabidopsis* homolog of *StRem1.3* (Raffaele et al., 2007). For these experiments, full length YFP was used to join VAM7-PX with AtRem1.4. Membrane patches, ranging in size from large to small punctae were observed upon expression of VAM7-PX-YFP-AtRem1.4 under the control of the *CaMV35S* promoter. However, constructs containing the *AtRem1.4* promoter uniformly produced small punctae (Supplementary Figure S4B). Patches produced by VAM7-PX-YFP-AtRem1.4 under the control of either promoter were abolished when the VAM7-PX^*^ mutant was used; instead, uniform PM binding characteristic of AtRem1.4 was observed with these mutant constructs (Supplementary Figures S4B,C). The transcript levels produced by the fusion constructs were validated by using quantitative real-time PCR, which showed transcript levels from the *CaMV35S* promoter to be ~6-fold higher than that from the *AtRem1.4* promoter (Supplementary Figure S4D). Thus, these results supported that the sizes of the patches were influenced by transgene expression levels. Furthermore, VAM7-PX-YFP-AtRem1.4 driven by the *AtRem1.4* promoter in stable transgenic *Arabidopsis* lines also resulted in uniform small punctae (Supplementary Figure S4E), indicating that this distribution on the plasma membrane was characteristic of the expression level produced by the *AtRem1.4* promoter.

### Formation of Membrane Patches Requires a PM-Binding Partner

StRem1.3 is exclusively targeted to the PM through a short C-terminal anchor which has been identified as an amphipathic α-helix (Perraki et al., 2012), or as an unconventional lipid-binding motif (Gronnier et al., 2017). Therefore, StRem1.3 is a typical peripheral membrane protein. To test if this membrane binding motif was required for formation of the membrane patches, mutations were introduced into this domain of StRem1.3. The mutant, StREM1.3^*^, showed only cytoplasmic localization (Supplementary Figure S5). When StREM1.3^*^ was paired with the VAM7-PX or Hrs2xFYVE PtdIns(3)P biosensors, the BiFC complexes showed only localization characteristic of the PtdIns(3)P biosensors (Supplementary Figure S5). Thus, membrane binding by StRem1.3 was required for formation of the patches.

**Figure 2:**
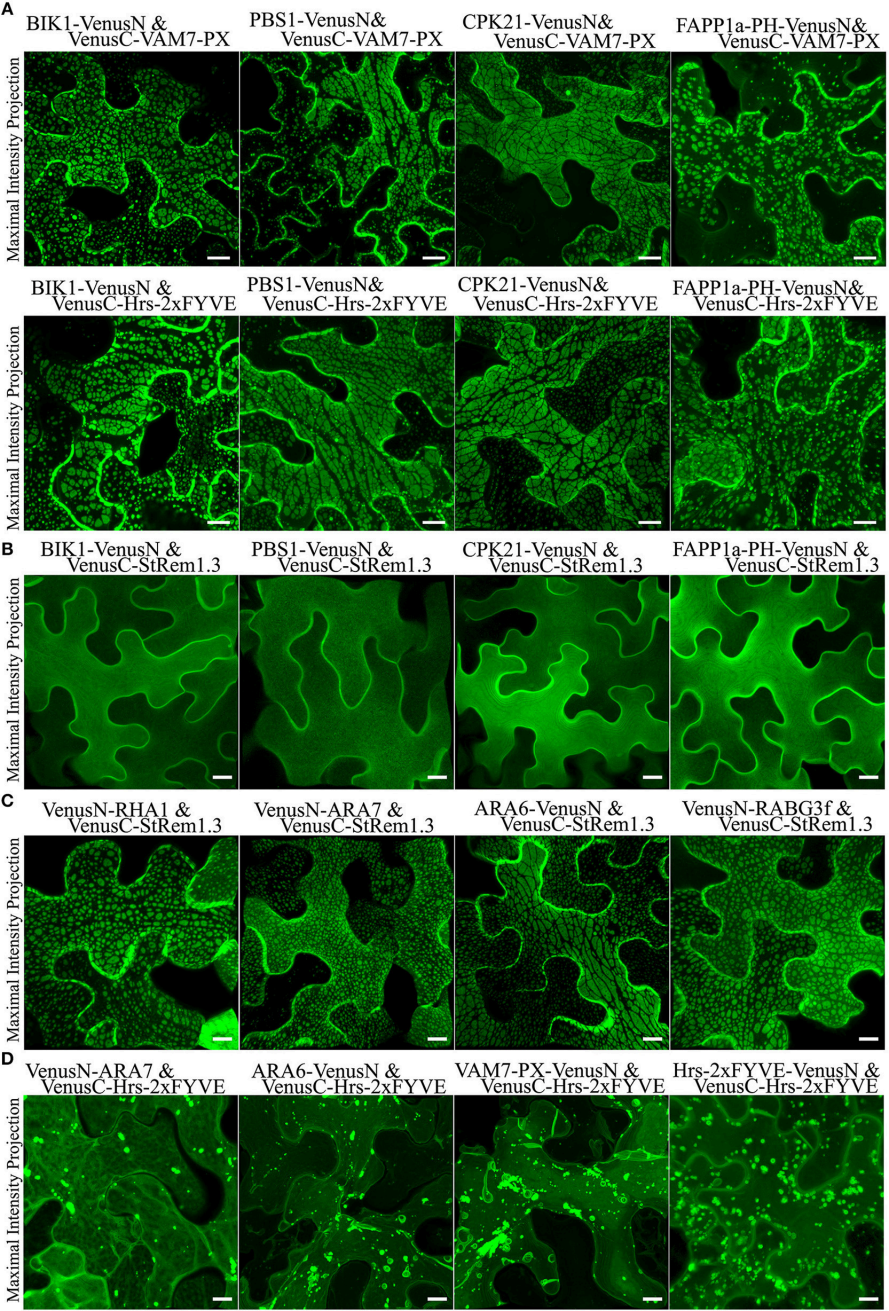
Membrane patches can be formed in *N. benthamiana* leaf cortical cells only by BiFC complexes containing a protein targeted to the PM and a protein targeted to multi-vesicular bodies (MVBs) and the tonoplast. **(A)** Peripheral membrane proteins BIK1, PBS1, CPK21, or PtdIns(4)P-binding protein FAPP1a fused to VenusN could produce membrane patches when co-expressed in BiFC complexes with VenusC-VAM7-PX or VenusC-Hrs-2xFYVE. **(B)** Fluorescence distribution of BiFC complexes containing only PM-targeted peripheral membrane proteins (PMPs). VenusN-fused BIK1, PBS1, CPK21, FAPP1a-PH were paired with VenusC-StRem1.3, as indicated. **(C)** Fluorescence distribution of BiFC complexes containing one PMP targeted to the PM and one PMP targeted to MVBs and the tonoplast. VenusN-fused RHA1, ARA7, ARA6, or RABG3f were paired with VenusC-StRem1.3 or FAPP1a-PH-VenusC, as indicated. **(D)** Fluorescence distribution of BiFC complexes containing only PtdIns(3)P biosensors and peripheral membrane proteins targeted to MVBs and the tonoplast. VenusC-Hrs-2xFYVE was paired with VenusN-fused ARA7, ARA6, VAM7-PX, or Hrs-2xFYVE. Scales are identical in all panels, representing 10 μm.

To test if other peripheral membrane proteins could also produce large membrane patches when paired with PtdIns(3)P biosensors, we replaced StRem1.3 with the well-characterized receptor-like cytoplasmic kinases (RLCKs), BIK (Lu et al., 2010), PBS1 (Qi et al., 2014), and CPK21 (Asai et al., 2013); those proteins are targeted to the PM via N-terminal myristoylation, palmitoylation, or both (Supplementary Figure S5). Consistent with the pattern observed with StRem1.3, when each of them was co-expressed with either VAM7-PX or Hrs-2xFYVE, fluorescent BiFC complexes distributed in large membrane patches were produced (Figure 2A). Additionally, when the myristoylation and palmitoylation sites of BIK1 were eliminated by mutation, the resultant BIK1^*^ mutant was not membrane localized and did not produce patches when paired with VAM7-PX or Hrs-2xFYVE (Supplementary Figure S5).

PtdIns(4)P accumulates on the PM of plant cells (Vermeer et al., 2009; Simon et al., 2016). Consistent with these reports, we observed that biosensors containing the PtdIns(4)P-binding PH domain of FAPP1 (Dowler et al., 2000) and Osh2p (Roy and Levine, 2004) were targeted to the PM (Supplementary Figure S5). In our experiments, we used a mutant of FAPP1-PH, namely FAPP1a-PH, that no longer binds the Golgi protein ARF1 and therefore cleanly detects PtdIns(4)P (He et al., 2011). When we paired these two PM-targeted PtdIns(4)P-binding proteins with either VAM7-PX or Hrs-2xFYVE, the resultant fluorescent BiFC complexes also appeared as large membrane patches similar to those produced with StRem1.3 (Figure 2A and Supplementary Figure S5). As a control, we used a FAPP1a-PH mutant, FAPP1a-PH^*^, in which the PtdIns(4)P binding site was abolished via site-directed mutagenesis (He et al., 2011). This mutant showed only cytoplasmic localization, resulting in complete loss of PM localization (Supplementary Figure S5). When paired with VAM7-PX and Hrs-2xFYVE, the FAPP1a-PH^*^ BiFC complexes displayed only organellar localization characteristic of the two PtdIns(3)P biosensors (Supplementary Figure S5). Together the above observations indicated that formation of membrane patches by PtdIns(3)P-binding BiFC complexes requires a peripheral membrane protein as a PM-binding partner.

### Membrane Patches Appear to Correspond to Tethering of the Tonoplast and MVBs to the PM

Based on the observations presented above, we formulated two alternative hypotheses regarding the origin of the membrane patches. The first hypothesis was that the patches resulted from aggregation of lipid microdomains, triggered by cross-linking of proteins or lipids enriched in those microdomains. The second hypothesis was that the patches were produced by the tethering to the PM of organelles such as endosomes, multi-vesicular bodies (MVBs) or the tonoplast that carried PtdIns(3)P in their membranes. The possibility that organellar tethering might be responsible for formation of the patches was suggested by our recent work (Tao et al., 2019) demonstrating that ER-PM tethering, analogous to that produced by *Arabidopsis* synaptotagmin1 (SYT1) (Yamazaki et al., 2010), could be produced by BiFC complexes carrying a PM-specific peripheral membrane protein and an ER-trafficked integral membrane protein (IMP).

To test the hypothesis that BiFC complexes triggered aggregation of membrane microdomains, we paired StRem1.3 with peripheral membrane proteins (PMPs) or PtdIns(4)P-binding proteins to examine whether they could trigger the formation of patches. All BiFC complexes involving two peripheral membrane proteins, namely BIK1-VenusN plus VenusC-StRem1.3, PBS1-VenusN plus VenusC-StRem1.3, CPK21-VenusN plus VenusC-StRem1.3, and FAPP1a-PH-VenusN plus VenusC-StRem1.3, were found to be homogeneously distributed on the PM (Figure 2B), closely similar to distribution characteristics of each protein fused with full-length FPs (Supplementary Figure S5). Thus, there was no evidence that cross-linking different PM-targeted PMPs in BiFC complexes could trigger the formation of patches.

To test the hypothesis that the BiFC complexes triggered tethering of PtdIns(3)P-containing membranes to the PM, we paired StRem1.3 with proteins that have been well-characterized as associating with MVBs and the tonoplast in *Arabidopsis*, namely the Rab5-type GTPases RHA1 (Sohn et al., 2003), ARA7 (Bottanelli et al., 2012), ARA6 (Ebine et al., 2011), and the Rab7-type GTPase RABG3f (Cui et al., 2014) (Supplementary Figure S6). Co-expressing those proteins with StRem1.3 in Venus BiFC complexes produced the characteristic membrane patches in every case (Figure 2C), supporting the hypothesis that this characteristic structure may correspond to the tethering of MVBs and/or the tonoplast to the PM.

To further examine whether the BiFC complexes may also connect the tonoplast to the PM, we used two well-identified tonoplast-localized proteins namely DUF679 membrane protein (AtDMP1) (Kasaras et al., 2012) and tonoplast potassium channel protein AtTPK1 (Maîtrejean et al., 2011), which are integral membrane proteins. When we co-expressed the two proteins with StRem1.3 however, the BiFC complexes produced ER-PM tethering, due to trapping of the two integral membrane proteins in the ER, as described in our work on ER-PM tethering (Tao et al., 2019).

When we paired a PtdIns(3)P biosensor with an MVB-associated protein, specifically VenusN-ARA7 plus VenusC-Hrs-2xFYVE or ARA6-VenusN plus VenusC-Hrs-2xFYVE, the BiFC complexes did not produce membrane patches (Figure 2D). BiFC complexes containing two different PtdIns(3)P biosensors, specifically VAM7-PX-VenusN plus VenusC-Hrs-2xFYVE or Hrs-2xFYVE-VenusN plus VenusC-Hrs-2xFYVE, also did not produce patches (Figure 2D). From this observation we concluded that, unlike PtdIns(4)P, PtdIns(3)P did not reside on the cytoplasmic face of the PM, and thus was not available to tether the PM to the tonoplast or MVBs.

### Both the Tonoplast and MVBs Can be Tethered to the PM

**Figure 3:**
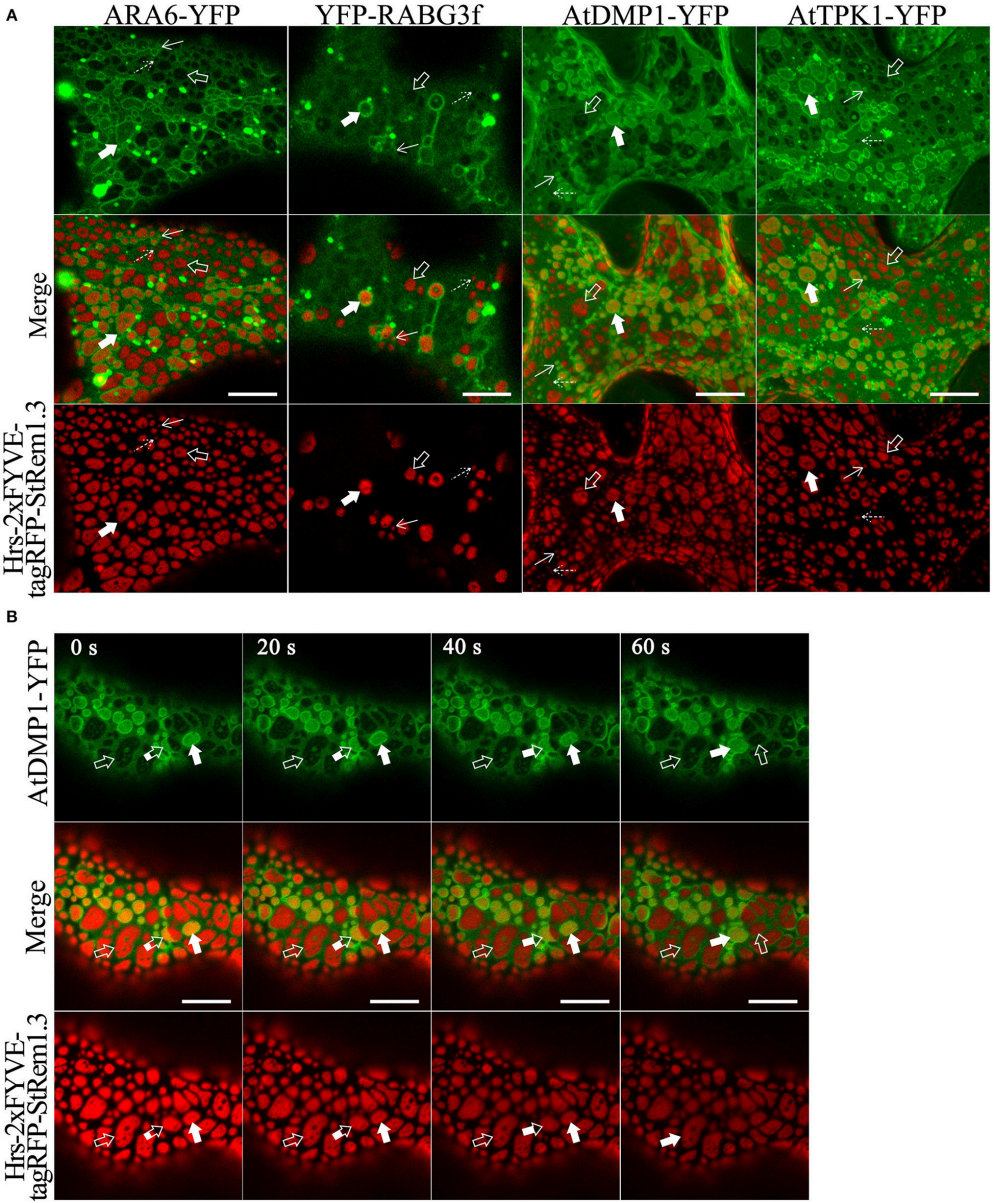
Membrane patches can involve PM-tethering of either the tonoplast or MVBs. **(A)** Distinct tonoplast- and MVB-associated patches revealed by co-expression of YFP- or GFP-fused ARA6, RABG3f, AtDMP1, or AtTPK1 with Hrs-2xFYVE-tagRFP-StRem1.3. Examples of tonoplast- and MVB-associated patches are highlighted with open and filled arrows, respectively. Punctae associating with the tonoplast or MVBs, are highlighted by dotted and solid arrows, respectively. **(B)** Dynamic nature of interactions of patches with MVBs revealed by time-lapse imaging of AtDMP1-GFP co-expressed with Hrs-2xFYVE-tagRFP-StRem1.3. Examples of patches that are tonoplast-associated, MVB-associated, or dynamically associated are highlighted with open, fully filled and partially-filled arrows, respectively. Animation of this cell is shown in Supplementary Movie S1. Scale bar in all panels represents 10 μm.

Since many marker proteins are shared between MVBs and the tonoplast, it was initially ambiguous whether tethering of the tonoplast, MVBs, or both were responsible for the formation of membrane patches. To examine the relationship of the patches to the MVBs and the tonoplast, we fused full length YFP to the MVB markers RHA1, ARA7, ARA6, and RABG3f, then co-expressed each of the fusions in turn with Hrs-2xFYVE-tagRFP-StREM1.3. We also co-expressed Hrs2xFYVE-tagRFP-StRem1.3 together with GFP fused to the tonoplast-markers AtDMP1 (Kasaras et al., 2012) or AtTPK1 (Maîtrejean et al., 2011). With both the MVB and tonoplast markers, we observed two patterns of interaction between the patches produced by Hrs-2xFYVE-tagRFP-StRem1.3 and the membranes stained with the respective GFP fusions. The GFP/YFP fusions labeled two sets of membranes (Figures 3A,B and Supplementary Figure S7). One set membranes, which we identified as the tonoplast, was moderately stained by the GFP or YFP markers and was spread over the entire width of the cell with wrinkling patterns in the 3D visualizations corresponding to furrows and ridges in the tonoplast (Supplementary Figure S7) as described previously (Marty, 1999; Reisen et al., 2005). In the tonoplast regions, the patches produced by Hrs-2xFYVE-tagRFP-StRem1.3 appeared to exclude much of the tonoplast-targeted fusion proteins (highlighted by open arrows in Figures 3A,B and Supplementary Figure S7), supporting that the BiFC complexes were embedded in the tonoplast and perhaps were forming aggregations that could exclude other tonoplast proteins. The second set of membranes, which we identified as MVBs, appeared as brightly stained complexes of tubes or vesicles; in these regions, the MVBs appeared focused on the patches, resulting in patches that appeared ringed by brightly stained MVB membranes (Figures 3A,B and Supplementary Figure S7). Some MVBs appeared highly dynamic (highlighted by partially filled arrows in Figure 3B, and displayed in Supplementary Movie S1), while others appeared more stably associated with patches (highlighted by filled arrows). Thus, we inferred that in these regions, the MVBs were tethered to the PM through the aggregations of Hrs-2xFYVE-tagRFP-StRem1.3 fusion proteins.

**Figure 4:**
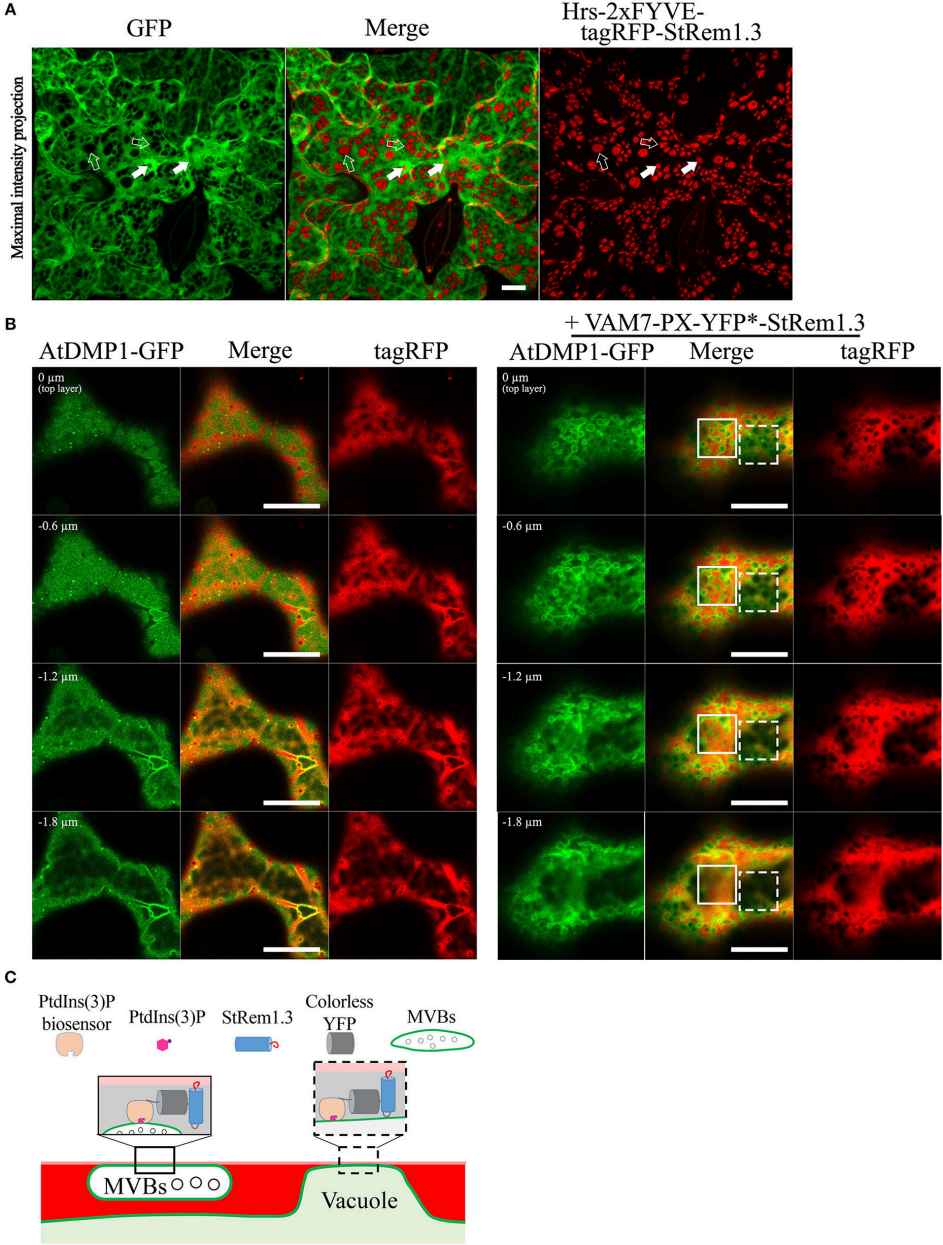
Distribution of cytoplasm-targeted free fluorescent proteins in the presence of membrane patches. **(A)** Soluble GFP showing exclusion by membrane patches produced by Hrs-2xFYVE-tagRFP-StRem1.3 (negatively stained holes in GFP panel). Examples of tonoplast-associated patches from which GFP has been excluded are highlighted with open arrows, and punctae are highlighted by dotted arrows. Examples of MVB-associated patches from which GFP has not been excluded are highlighted with filled arrows, and punctae are highlighted by solid arrows. **(B)** Left panel: Serial *z*-axis sections of control cells co-expressing tagRFP and AtDMP1-GFP Right panel: Serial z-axis sections of cells co-expressing tagRFP and AtDMP1-GFP together with the fusion protein VAM7-PX-YFP^*^-StRem1.3 that produces colorless, negatively-stained patches. The sections reveal that the cytoplasm overlaps patches associated with MVBs, but not with patches associated with the tonoplast. Examples of tonoplast- and MVB-associated patches are highlighted with dotted and solid squares, respectively. The Z-axis image scanning interval was 0.6 μm. Animations of serial *z*-sections shown in the left and right panels can be viewed in Supplementary Movies S2, S3, respectively. **(C)** An explanatory model for the distribution patterns of soluble cytosol, the vacuole, tonoplast, and MVBs in the presence of colorless membrane patches as observed in the right panel of **(B)**. Explanations of tonoplast- and MVB-associated patches are highlighted with dotted and solid squares, respectively. Scale bars in **(A,B)**, represent 10 μm in each case.

To further test these inferences, we co-expressed soluble GFP with Hrs-2xFYVE-tagRFP-StRem1.3, to determine if the cytoplasm was displaced, as expected if the tonoplast was tethered directly to the PM. As shown in Figure 4A, the freely diffusing cytoplasmic GFP proteins were clearly excluded by many of the patches. To further examine the inferred tethering of the MVBs, we co-expressed soluble tagRFP and the tonoplast marker, AtDMP1-GFP, together with VAM7-PX-YFP^*^-StRem1.3. VAM7-PX-YFP^*^-StRem1.3 contains a colorless mutant of YFP (Stepanenko et al., 2011) and thus produces colorless patches that can be visualized by negative staining with proteins that they exclude such as tagRFP and AtDMP1-GFP. As shown in Figures 4B,C and Supplementary Movies S2, S3, the tagRFP fluorescence was substantially excluded by the patches in regions lacking MVBs (highlighted by the white rectangle with a dashed border). However, in the regions containing MVBs, tagRFP fluorescence appeared excluded in some *z*-sections, but not in other *z*-sections of the same region (highlighted by the white rectangle), indicating that the tonoplast was not closely appressed to the PM in those regions and that a layer of cytoplasm covered the MVBs associated with the patches. We also note that the exclusion of AtDMP1-GFP and tagRFP by the colorless VAM7-PX-YFP^*^-StRem1.3 rules out that the exclusion phenomenon is an artifact of confocal microscopy in regions expressing two different fluorescent proteins.

### PM-MVB/TP Tethering Also Modifies the Distribution of PM-Localized Proteins

**Figure 5:**
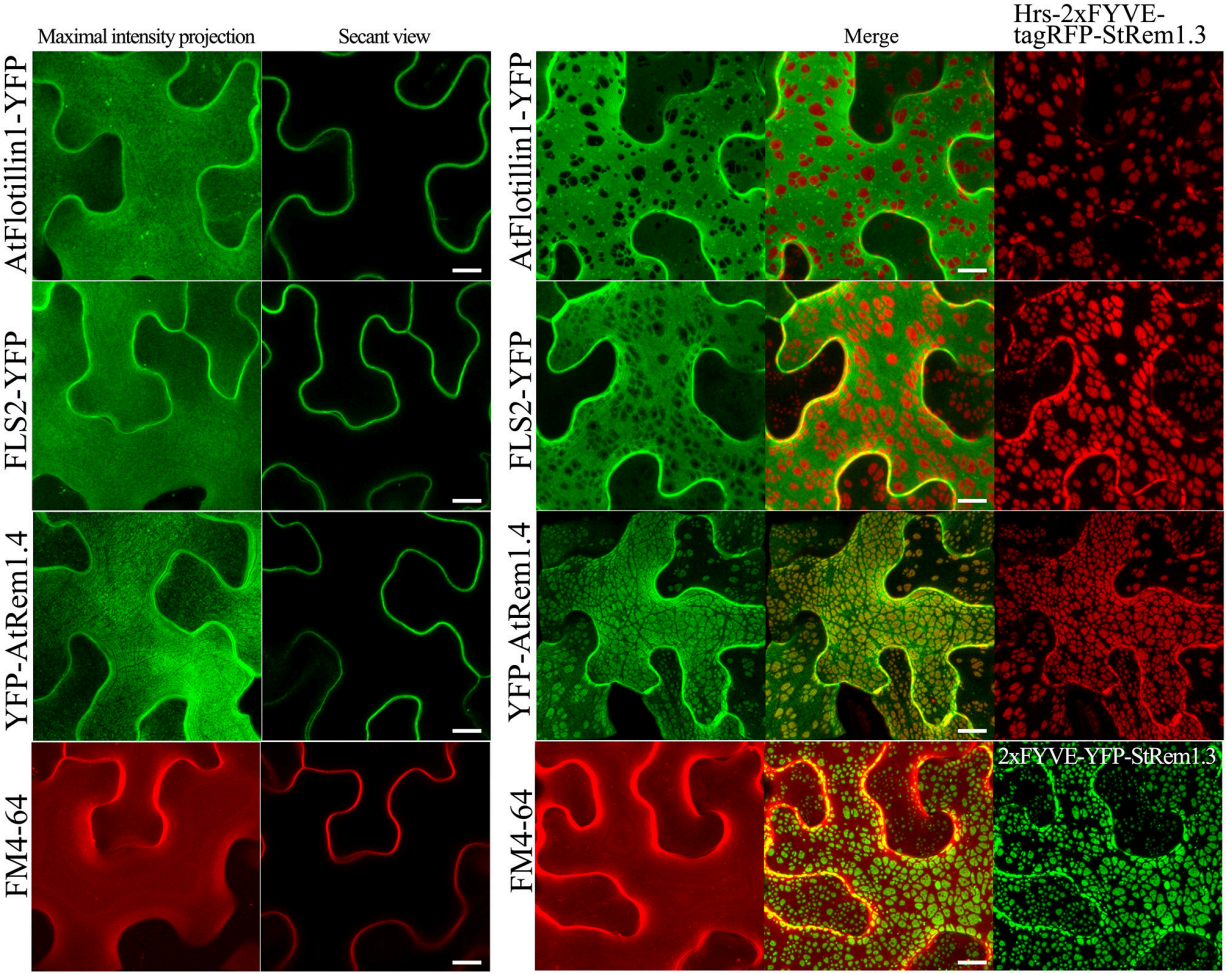
Distribution of fluorescently tagged PM-localized proteins in the presence of membrane patches in *N. benthamiana* leaf cortical cells. Left panels: the distribution of PM-targeted proteins expressed alone, including YFP-tagged membrane microdomain protein AtFlotillin1, integral membrane protein FLS2, and AtRem1.4 which is the closest *Arabidopsis* homolog of StRem1.3. Right panels: the distribution of those fluorescently tagged PM proteins co-expressed with Hrs-2xFYVE-tagRFP-StRem1.3. Bottom panels: FM4-64 staining in the presence or absence of expressed Hrs-2xFYVE-YFP-StRem1.3. All scale bars represent 10 μm.

In our ER-PM tethering study (Tao et al., 2019), we showed that the membrane microdomain-associated peripheral membrane protein, AtFlotillin1, was spatially excluded from the regions of the PM involved in ER-PM tethering produced by BiFC complexes. To determine whether PM-MVB/TP junctions could also modify the distribution of PM proteins, we co-expressed Hrs-2xFYVE-tagRFP-StRem1.3 fusion proteins with AtFlotillin1. We observed that the patches corresponding to the PM-MVB/TP tethering regions substantially excluded the co-expressed AtFlotillin (Figure 5). For the PM-located transmembrane receptor kinase FLS2 (Gómez-Gómez and Boller, 2000), a similar spatial exclusion was also observed (Figure 5). As a control, we stained the PM using the lipophilic styryl dye FM4-64 which intercalates into the outer leaflet of the PM (Dupont et al., 2010; Song et al., 2012). No exclusion of the FM4-64 stain was observed (Figure 5). Interestingly, in contrast to the exclusion patterns observed for PM-associated proteins noted above, AtRem1.4, which could form hetero-oligomers with StRem1.3 (Marín et al., 2012; Perraki et al., 2012; Jarsch et al., 2014), was enriched at the PM-MVB/TP tethering sites produced by Hrs-2xFYVE-tagRFP-StRem1.3 (Figure 5).

In order to further verify that the regions of exclusion observed above were not created by a microscopy artifact, we again used the colorless fusion protein, VAM7-PX-YFP^*^-StRem1.3. As expected, the colorless patches of VAM7-PX-YFP^*^-StRem1.3 could also cause exclusion of AtFlotillin, and FLS2 (Supplementary Figure S8). Moreover, to test if the fluorescing patches could suppress fluorescence of the layer immediately below (Poteser et al., 2016), we co-expressed Hrs-2xFYVE-YFP-StRem1.3 with the vacuolar lumen marker SP-tagRFP-AFVY (Hunter et al., 2007). No depletion of SP-tagRFP-AFVY fluorescence was observed (Supplementary Figure S8).

### PM-MVB/TP Tethering Is Limited by the Cytoskeleton and ER Network

We noticed that the patches produced by produced by PM-TP/MVB tethering displayed a degree of order. For example, the observed patches often appeared to negatively stain long tracks (Supplementary Figure S9). To test the hypothesis that these tracks corresponded to cortical microtubules, we co-expressed the cortical microtubule (MT) marker *Arabidopsis* Casein Kinase 1-Like 6 (ACK6) (Ben-Nissan et al., 2008) together with Hrs-2xFYVE-YFP-StRem1.3. As shown in Supplementary Figure S9, it was clearly evident that many tethered patches were separated by microtubules. In some cases, growing microtubules could be observed dividing a patch into two smaller patches (highlighted by the empty arrow in Supplementary Figure S9). Similar results were also observed when the actin marker AtFimbrin1 (Wang et al., 2004) was co-expressed with Hrs-2xFYVE-YFP-StRem1.3, except that orthogonal imaging revealed that the actin cytoskeleton also formed a thin mesh layer beneath the patches (Supplementary Figure S9).

The cortical endoplasmic reticulum (ER) is a region of the ER closely juxtaposed to the PM in animal cells (Zhang et al., 2012) and plant cells (Sparkes et al., 2010; Stefano et al., 2014). Using the ER-marker SP-tagRFP-HDEL (Matsushima et al., 2002), we could observe the relationship between the PM-MVB/TP patches and the cortical ER. As shown in Supplementary Figure S9, the cortical ER and the patches appeared to be mutually exclusive, as expected if the patches represent sites of PM-MVB/TP tethering.

### PM-MVB/TP Tethering Is Associated With Two Plant U-box (PUB) Armadillo (ARM) Repeat E3 Ubiquitin Ligases

**Figure 6:**
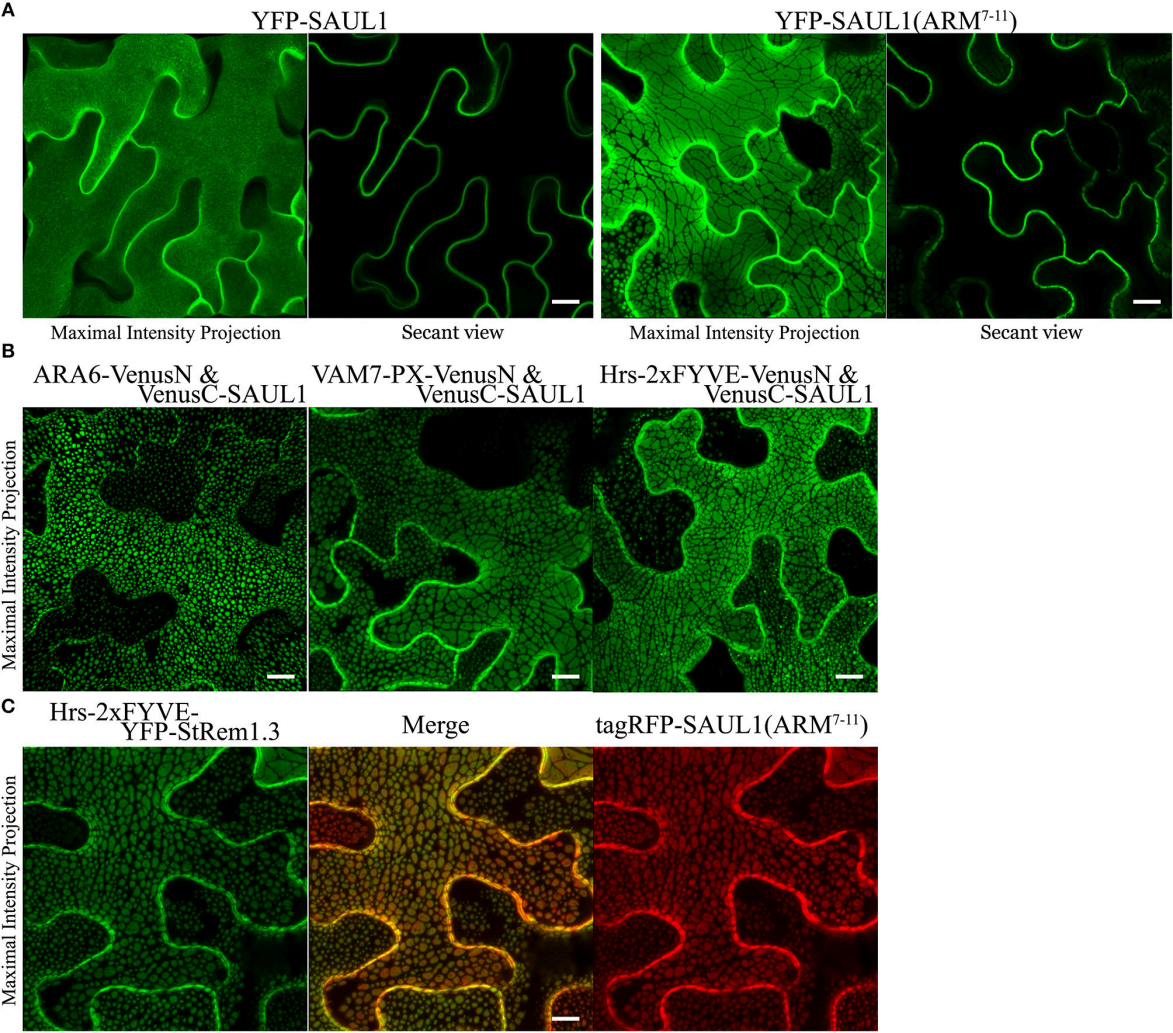
Association of membrane patches with E3 ubiquitin ligase SAUL1 in *N. benthamiana* leaf cortical cells. **(A)** Subcellular localization of YFP-tagged full-length SAUL1 and YFP-tagged SAUL1 C-terminal ARM repeats 7-11. **(B)** The full-length SAUL1 co-expressed with either ARA6, or VAM7-PX, or Hrs-2xFYVE in BiFC complexes forms patches indicative of PM-MVB/TP tethering. **(C)** Co-localization of membrane patches created by expression of Hrs-2xFYVE-YFP-StRem1.3 and patches created by expression of tagRFP-SAUL1(ARM^7−11^). All scale bars are representing 10 μm.

Drechsel et al. (2010) reported that *Arabidopsis* PUB (plant U-box) E3 ligase senescence-associated ubiquitin ligase 1 protein (SAUL1, also called AtPUB44) was exclusively targeted to the PM with a homogenous distribution. However, when its C-terminal ARM domain (repeats 7–11), was expressed as a YFP fusion protein, a heterogeneous pattern of large membrane patches was observed, similar to the patches we have described here. A similar result was also reported for its closest paralog AtPUB43 (Vogelmann et al., 2014). To investigate whether PM-MVB/TP tethering was associated with the SAUL1 and AtPUB43 patches, we carried out subcellular co-localization experiments. When wild type SAUL1 was fused with YFP at the N terminus and transiently expressed in *N. benthamiana* cortical cells, the fluorescence of YFP-SAUL1 was uniformly distributed on the PM (Figure 6A) consistent with the previous studies (Drechsel et al., 2010; Vogelmann et al., 2014). Furthermore, expressing YFP-tagged ARM repeats 7–11 of SAUL1 produced fluorescent signals distributed into patches (Figure 6A), again as previously observed (Drechsel et al., 2010; Vogelmann et al., 2014). Interestingly, when we co-expressed full-length SAUL1 with ARA6, VAM7-PX, or Hrs-2xFYVE in BiFC complexes, we also observed patches characteristic of PM-MVB/TP tethering, suggesting that SAUL1 could indeed participate in tethering (Figure 6B). When we expressed Hrs-2xFYVE-YFP-StRem1.3 to produce PM-MVB/TP tethering and at the same time co-expressed tagRFP-SAUL1(ARM^7−11^), the patches produced by both fusion proteins fully coincided (Figure 6C) suggesting that both fusion proteins produced tethering by the same mechanism. Similar results were also observed with AtPUB43 (Supplementary Figure S10). We also observed complete overlap between patches produced by YFP-SAUL1(ARM^7−11^) and tagRFP-AtPUB43(ARM^7−11^), and between patches produced by Hrs-2xFYVE-YFP-StRem1.3 and tagRFP-AtPUB43(ARM^7−11^) (Supplementary Figure S10) suggesting that AtPUB43 could produce tethering by the same mechanism.

**Figure 7:**
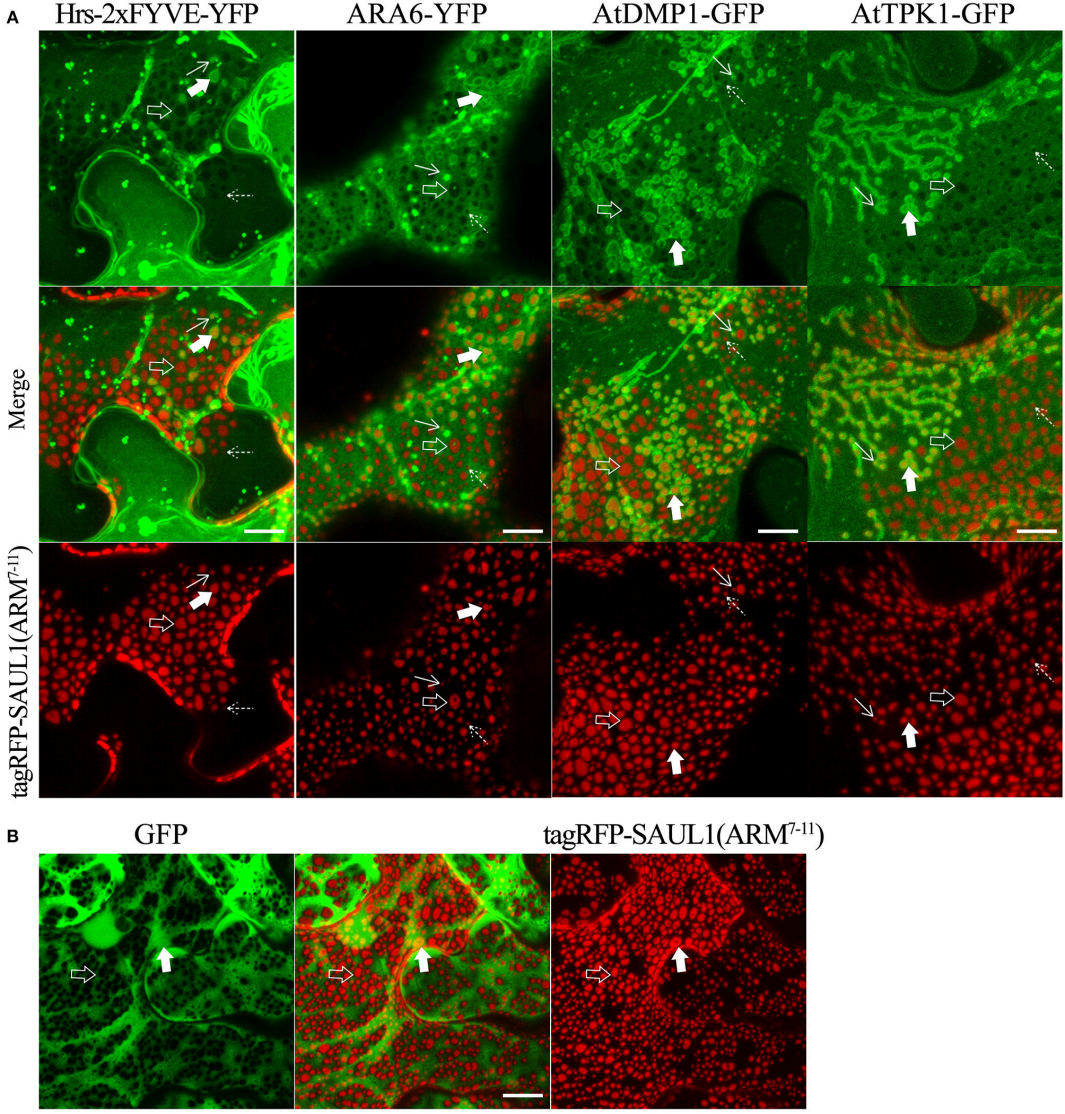
Distribution of fluorescent tonoplast, MVB and cytoplasmic marker proteins in the presence of membrane patches created by expression of tagRFP-SAUL1(ARM^7−11^) in *N. benthamiana* leaf cortical cells. **(A)** Distinct tonoplast- and MVB-associated patches revealed by co-expression of YFP- or GFP-fused Hrs-2xFYVE, ARA6, AtDMP1 or AtTPK1 with tagRFP-SAUL1(ARM^7−11^). Examples of tonoplast- and MVB-associated patches are highlighted with open and filled arrows, respectively. Similarly, punctae associating with the tonoplast or MVBs, are highlighted by dotted and solid arrows, respectively. **(B)** Distribution of cytoplasmic GFP co-expressed with tagRFP-SAUL1(ARM^7−11^). Examples of tonoplast-associated patches from which GFP has been excluded are highlighted with open arrows while MVB-associated patches from which GFP has not been excluded are highlighted with filled arrows. All scale bars are representing 10 μm.

To more directly test whether the SAUL1 and AtPUB43 patches represented PM-MVB/TP tethering, we co-expressed tagRFP-SAUL1(ARM^7−11^) with fluorescently tagged Hrs-2xFYVE, ARA6, AtDMP1, or AtTPK1 to label the MVBs and TP. In each case, patches characteristics of tethering to both the TP (highlighted by the open arrows in Figure 7A) and to the MVBs (highlighted by the filled arrows in Figure 7A) were observed. These results supported that SAUL1(ARM^7−11^) alone could produce PM-MVB/TP tethering, suggesting that the protein has the ability to bind both the PM and also to the TP or MVBs. Similar results were also observed with tagRFP-AtPUB43(ARM^7−11^) (Supplementary Figure S11). The SAUL1 and AtPUB43 patches also exhibited exclusion of soluble GFP (Figure 7B and Supplementary Figure S11), confirming tethering to the tonoplast.

**Figure 8:**
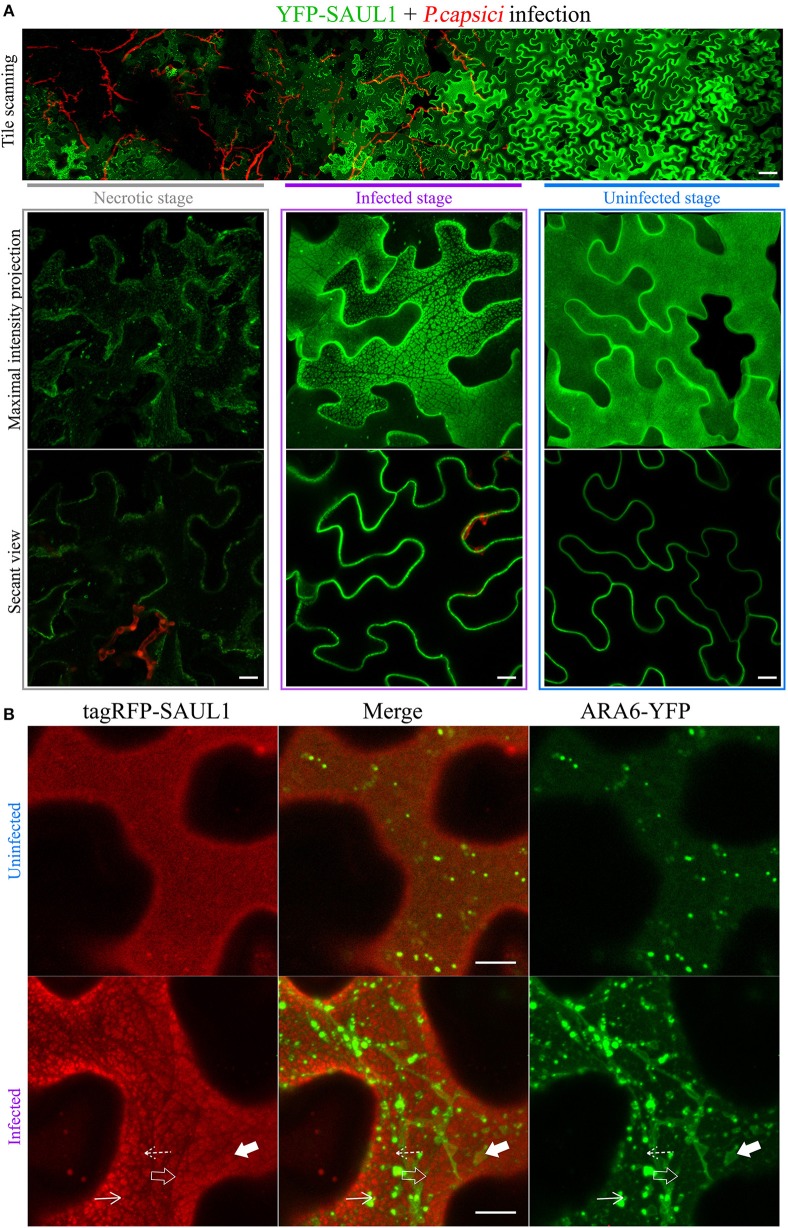
Distribution of the fluorescently tagged full-length SAUL1 in *N. benthamiana* leaf cortical cells during pathogen infection. **(A)** Cortical cells expressing YFP-tagged SAUL1 were infected by GFP-tagged oomycete *P. capsici* (artificially represented by the red color). Top panel: tile-scan imaging showing the changing distribution of YFP-SAUL1 on the PM associated with the progress of *P. capsici* infection. Scale bar in this panel represents 50 μm. Lower panel: details of the distribution of YFP-SAUL1 in the necrotic (outlined by gray box), infected (outlined by purple box) and uninfected cells (outlined by blue box). **(B)** Distribution of ARA6-YFP compared to tagRFP-SAUL1 in uninfected and infected cells. The tonoplast- and MVB-associated patches formed by YFP-SAUL1 were revealed by ARA6-YFP as highlighted with open and filled arrows, respectively. Similarly, punctae associating with the tonoplast or MVBs, are highlighted by dotted and solid arrows, respectively. All other scale bars represent 10 μm.

Since full length SAUL1 (AtPUB44) and AtPUB43 were uniformly distributed on the PM, but their isolated ARM repeats^7−11^ could produce PM-MVBs/TP tethering, we hypothesized that there may be conditions when full-length SAUL1 and AtPUB43 may naturally regulate TP/MVBs-PM tethering. To explore this hypothesis, we examined the subcellular distribution of full-length SAUL1 and AtPUB43 in the context of infection, based on two rationales. First, SAUL1 and AtPUB43 have been reported as being redundantly involved in plant defense (Disch et al., 2015; Tong et al., 2017). Second, MVBs have been identified as a potential source of exosomes (Colombo et al., 2014), which may be associated with plant defense against pathogen infection (An et al., 2007; Samuel et al., 2015; Rutter and Innes, 2017). Therefore, we inoculated YFP-SAUL1-expressing or YFP-AtPUB43-expressing leaves with the oomycete pathogen *P. capsici* LT263, using a transformant that constitutively expressed GFP (represented by the artificial red color in Figure 8A and Supplementary Figure S12). At 24–36 h post-inoculation, the distribution of YFP-SAUL1 was observed across the margin of the pathogen lesion using tile scanning combined with 3D projection. In this way, the spatial and temporal development of infection could be recorded in a single composite image with high resolution (top panel in Figure 8A). In cells that were located in regions not infiltrated with hyphae (uninfected stage in Figure 8A), YFP-SAUL1 was homogeneously distributed on the PM. In tissue that was in the process of being invaded by the *P. capsici* hyphae (infected stage in Figure 8A), YFP-SAUL1 formed heterogeneous membrane patches on many of the cells. At the later stages of infection, where dying host cells displayed membrane rupture and cell shrinkage, little YFP-SAUL1 could be visualized. When co-expressed with ARA6-YFP, the membrane patches labeled by tagRFP-SAUL1 during the infection were either ringed by ARA6-YFP-labeled MVBs (highlighted by the filled arrows in Figure 8B) or excluded the ARA6-YFP on the tonoplast (highlighted by the open arrows in Figure 8B). Similar results were obtained with full-length AtPUB43 (Supplementary Figure S12). Taken together, these results suggested that SAUL1 and AtPUB43 might be associated with PM-MVB/TP tethering in plants during infection.

## Discussion

In this study, in the process of mapping the distribution of PtdIns(3)P relative to plasma membrane (PM) markers, we observed that BiFC complexes containing a PtdIns(3)P-binding protein, such as VAM7-PX or Hrs-2xFYVE, together with a PM-localized peripheral membrane protein such as the remorin protein StRem1.3 or BIK1, were distributed in large but heterogeneous PM patches (Figures 1, 2). Proteins that localized to the PM via PtdIns(4)P-binding, such as FAPP1a-PH and Osh2p, could also participate in the formation of the patches. Control experiments with mutant proteins confirmed that both PtdIns(3)P-binding and PM-binding by the respective partners were essential for the formation of these patches. By analogy with our recent discovery that BiFC complexes could produce ER-PM tethering (Tao et al., 2019), we hypothesized that BiFC complexes that combine PM-binding with PtdIns(3)P-binding might produce tethering of the tonoplast and MVBs to the PM. We found support for this hypothesis by showing that MVB-associated proteins such as Rab5-type GTPases RHA1, ARA6, and ARA7 as well as Rab7-type GTPase RABG3f could all induce the observed patches when partnered with StRem1.3 in BiFC complexes (Figure 2). We observed that all of these MVB-associated proteins also stained the tonoplast, albeit at lower intensity, as previously reported for ARA6 and ARA7 (Ebine et al., 2011). An alternative hypothesis, that the BiFC complexes triggered aggregation of lipid micro-domains, was not supported by these observations. In particular, the ability of the observed patches to exclude cytoplasmic markers was consistent with TP tethering, but not with aggregation of lipid microdomains. As in the case of ER-PM tethering (Tao et al., 2019), PM-MVB/TP tethering could restrict the distribution of PM-associated and tonoplast-associated proteins. Importantly, we found that the heterogeneous pattern of membrane patches, reported for two *Arabidopsis* PM-associated U-box(PUB) E3 ubiquitin ligases, namely SAUL1 and its closest paralog AtPUB43 (Drechsel et al., 2010; Vogelmann et al., 2014), also appeared to represent PM-MVB/TP tethering, suggesting SAUL1 and AtPUB43 may function as natural tethers under certain conditions.

In eukaryotic cells, membrane-bound organelles are generally segregated to support their individual cellular functions. However, functional communications among organelles may occur via vesicular transport, most notably in the secretory and endosomal trafficking pathways (Bonifacino and Glick, 2004). Alternatively, appositions between two organelles, often referred to as membrane contact sites (MCSs) may enable inter-organellar communication (Prinz, 2014). MCSs are stabilized by multi-domain tethering proteins which can bridge the membranes of two organelles without promoting their fusion (Helle et al., 2013; Prinz, 2014; Islinger et al., 2015). To date, specific tether proteins have been characterized for MCSs between ER and endosomes/lysosomes/vacuoles/MVBs, ER and mitochondria, ER and peroxisomes, ER and Golgi, ER and chloroplasts, ER and phagosomes, Golgi and Golgi, PM and ER, and PM and mitochondria (Prinz, 2014; Eden, 2016). However, to date there have been no reports of tethering proteins that naturally establish MCSs between the PM and MVBs or the tonoplast. Here, our observations of SAUL1 and its paralog AtPUB43 (Figure 6 and Supplementary Figure S10) suggest that under some circumstances they might function as tethers, mediating docking of the tonoplast and MVBs to the PM. Not only could the full length SAUL1 and AtPUB43 proteins promote tethering in cells within oomycete-infected tissue, but the C-terminal membrane binding domain (ARM7–11) of each protein could also promote tethering when expressed alone. Also, fusion of SAUL1 or AtPUB43 to ARA6 via BiFC complexes also produced tethering. Thus, we speculate that there may be regulatory events such as phosphorylation or ligand binding that might modify the full-length proteins to allow their C-terminal domains to bind to MVBs and the tonoplast (or proteins within them, such as ARA6) as well as to the PM, to promote tethering. We also cannot currently rule out that the tethering associated with SAUL1 and AtPUB43 during infection was triggered by the pathogen as a virulence mechanism.

As a member of the PUB family of E3 ubiquitin ligases, SAUL1 has been reported to be involved in the regulation of senescence, cell death, and PAMP-triggered immunity (Drechsel et al., 2010; Disch et al., 2015; Tong et al., 2017). In contrast to other PUB E3 ligases, SAUL1 and its closest paralog, AtPUB43, are exclusively located on the PM (Drechsel et al., 2010; Vogelmann et al., 2014), which we confirmed in this study. SAUL1 and AtPUB43 carry ARM repeats, which are thought to mainly function as interfaces for protein-protein interactions (Coates, 2003) or lipid interactions (Antignani et al., 2015). Thus, it is plausible that ARM repeats 7–11 of SAUL1 and AtPUB43 might mediate membrane binding by these proteins, either through direct membrane interactions or through contacts with other membrane proteins. It remains unknown whether protein ubiquitination by the E3 ligase activities such as SAUL1 and AtPUB43 is required for tethering through regulation of membrane or vesicle interactions. In future, it will be of interest to map the potential interacting partners of SAUL1 and AtPUB43, especially partners that are located in the tonoplast and MVB membranes.

Docking of vesicles to the PM is a normal part of the secretion process, releasing cellular molecules, and regulating the composition of plasma membrane (Grant and Donaldson, 2009; Hsu and Prekeris, 2010; Donovan and Bretscher, 2015; Wu and Guo, 2015). These vesicles typically originate from the TGN or from recycling endosomes. In plants, TGNs function as early endosomes as well as the sorting hub for secretory vesicles (Scheuring et al., 2011; Paez Valencia et al., 2016). The targeting and tethering of secretory vesicles to the PM involves the octameric exocyst complex (Hála et al., 2008; Žárský et al., 2013). As noted in the introduction, an alternative secretion process that has gained renewed attention recently, especially in the context of plant-microbe interactions, is the release of exosomes (Regente et al., 2017; Rutter and Innes, 2018). Proteins involved in the biogenesis and secretion of exosomes in plants are not fully characterized. However, a recent proteomic analysis indicated that plant exosomes might share similar proteomes with other endomembrane organelles, including those containing Rab5-type GTPases or Rab7-type GTPases (Rutter and Innes, 2017). It is noteworthy that ARA6 has been identified as involved in membrane fusion of endosomes with the PM (Ebine et al., 2011). Moreover, ARA6 was found localized at the specialized extrahaustorial membrane (EHM) in *Arabidopsis* and barley when infected by powdery mildew fungi (Nielsen et al., 2012; Inada et al., 2016). ARA6 was reported as accumulating at fungal infection sites and being partially co-localized with the homolog of mammalian exosome membrane protein CD63 in *Arabidopsis* namely TETRASPANIN 8 (TET8) (Cai et al., 2018). ARA6 was also associated with PEN1 on the PM, and could promote VAMP727–PEN1 complex formation at the PM (Ebine et al., 2011). Therefore, ARA6 has been suggested as the most likely candidate mediating exosome release (Hansen and Nielsen, 2018). In this context, here we observed that ARA6 could be directly involved in the formation of PM-MVB tethering associated with SAUL1 or AtPUB43 during pathogen infection (Figure 8 and Supplementary Figure S12). The release of exosomes from MVBs presumably involves contact between the MVB membrane and the PM, followed by fusion of those membranes (Skotland et al., 2017). Our observation that SAUL1 and AtPUB43 can mediate PM-MVB tethering under certain circumstances (over-expression during infection, expression of the C-terminal ARM domain, fusion to MVB-bound proteins such as ARA6) raises the interesting question of whether one or both of these two E3 ligases may play a role in regulating exosome release. SAUL1 has been reported to be induced during infection and to be required for PAMP-triggered immunity (Tong et al., 2017). Our results also indicate that SAUL1 and AtPUB43 may be associated with tethering of the tonoplast to the PM. This raises the additional interesting question of whether the vacuole might be a direct source of exosomes under some circumstances. We observed that constructs driven by the native AtREM1.4 promoter produced small punctae, similar in size to ER-PM contact sites, both during *N. benthamiana* transient expression and in transgenic *Arabidopsis* plants. SAUL1 and AtPUB43 constructs also produced small punctae, in addition to the larger patches, including during infection. Future work will be needed to determine if these small punctae represent true PM-MVB/TP contact sites, and whether such contact sites might be involved in exosome release.

During plant-microbe interactions, effector proteins are secreted from microbial pathogens and enter host cells to manipulate host immune signaling responses to promote successful infection. However, questions about how effectors from fungi and oomycetes are translocated into host cells have not been fully resolved yet. Several studies have reported that pathogen effectors have the capability to bind PtdIns(3)P *in vitro* (Gan et al., 2010; Kale et al., 2010; Gu et al., 2011; Plett et al., 2011; Weigele et al., 2017). Therefore, it was proposed that PtdIns(3)P-binding may be involved in host entry by effectors, possibly via lipid microdomain-mediated endocytosis (Kale et al., 2010; Gu et al., 2011; Plett et al., 2011). This study was initiated with the aim of establishing more clearly whether PtdIns(3)P occurred on the cytoplasmic face of the PM. The results presented here reveal that PtdIns(3)P is present on a wide variety of vesicles, the MVB, and also the tonoplast. However, based on the inability of PtdIns(3)P-binding proteins to provide the PM attachment required for PM-MVB/TP tethering, in contrast to PtdIns(4)P-binding proteins, we could find no evidence for PtdIns(3)P occurring on the cytoplasmic face of the PM. This conclusion is in agreement with our work on ER-PM tethering (Tao et al., 2019), where it was shown that PtdIns(3)P-binding proteins could not provide the PM attachment required for ER-PM tethering, whereas proteins that bound PtdIns(4)P and PtdIns(4,5)P_2_ could do so. The possibility that PtdIns(3)P-bearing membranes may be involved in the release of exosomes suggests that the relationship between effector secretion and exosome release should be examined.

This study also further highlights the risks of using the BiFC assay to study protein-protein or protein-membrane interactions in plants, as the spontaneous formation of BiFC complexes could lead to alterations in cellular structure and membrane organization. At the same time, our results open the possibility of using tethering as a tool to explore or manipulate the arrangement of membrane proteins and organelles. For example, our observation that the patches of tethering can exclude or include different proteins and lipids on the PM and tonoplast may assist in distinguishing different membrane subdomains.

In summary, in this study and in Tao et al. (2019), we have developed an extensive set of tools for examining the spatial relationships of organelles, membranes, membrane proteins, and lipids relative to one another, including a set of mutant marker proteins for use as negative controls (summarized in Table 1 and Supplementary Table S1). We have demonstrated that the efficient spontaneous formation of BiFC complexes by some fluorescent proteins, such as Venus, can be exploited to artificially induce tethering of organelles and to more clearly infer the membrane localization of proteins and lipids. We showed how the tools could be used to identify the existence of PM-MVB/TP tethering under certain circumstances. At the same time, our study highlights the risks of altering cellular structures and membrane organization that researchers should be aware of when using the BiFC assay to study protein-protein or protein-membrane interactions in plants.

**Table 1:** Fluorescent marker proteins and mutants used in this study.

**Protein**	**ACCESSION/TAIR**	**Region**	**Localization in *N. benthamiana* leaf cortical cells**	**Features**	**References**
**SUBCELLULAR MARKERS**					
VAM7-PX	NP_011303	1–134	MVB, tonoplast	PtdIns(3)P binding	Kale et al., 2010
VAM7-PX^*^		1–134	Cytoplasm	Mutations in PtdIns(3)P binding site: R40E, S42A	Lee et al., 2006
Hrs-2xFYVE	NP_032270	147–223	MVB, tonoplast	Tandem repeat of PtdIns(3)P binding domain, linked by QGQGS	Vermeer et al., 2006
Hrs-2xFYVE^*^		147–223	Cytoplasm	Mutations in PtdIns(3)P binding site: R34S, K35S, H36S, H37S, R39S (both repeats)	Kutateladze and Overduin, 2001; Pankiv et al., 2010
StRem1.3	NP_001274989	FL	PM	PMP	Perraki et al., 2012
StRem1.3^*^		FL	Cytoplasm	Mutations of hydrophobic residues at the C terminus: L179H, A180H, A181H, Y184S, A185S, G187V, A189A, L194S, G195Q, I196Q, F197Q	Perraki et al., 2012
AtRem1.4	At5g23750	FL	PM	PMP	Raffaele et al., 2007
BIK1	At2g39660	FL	PM	PMP	Lu et al., 2010
BIK1^*^		FL	Cytoplasm (mainly), cortical microtubules	Mutation of palmitoylation site: G1A	Tao et al., 2019
PBS1	AT5G13160	FL	PM	PMP	Qi et al., 2014
CPK21	AT4G04720	FL	PM	PMP	Asai et al., 2013
FAPP1a-PH	AAG15199	1–99	PM	FAPP1-PH protein containing mutations of the ARF1 binding site: E50A, H54	He et al., 2011
FAPP1a-PH^*^		1–99	Cytoplasm	Mutations in PtdIns(4)P binding site: K7E, R18A	He et al., 2011
Osh2p	NM_001180078	256–424	PM	Tandem repeat of PtdIns(4)P binding domain, linked by QGQGS	Roy and Levine, 2004
ARA6	AT3G54840	FL	MVB (mainly), tonoplast	PMP	Ebine et al., 2011
RHA1	AT5G45130	FL	MVB (mainly), tonoplast	PMP	Sohn et al., 2003
ARA7	AT4G19640	FL	MVB (mainly), tonoplast	PMP	Bottanelli et al., 2012
RABG3f	AT3G18820	FL	MVB (mainly), tonoplast	PMP	Cui et al., 2014
AtDMP1	AT3G21520.1	FL	MVB, tonoplast (mainly)	IMP (GFP inserted between 108E and 109P)	Kasaras and Kunze, 2017
AtTPK1	AT5G55630	FL	MVB, tonoplast (mainly)	IMP	Maîtrejean et al., 2011
SAUL1	AT1G20780	FL	PM	PMP	Drechsel et al., 2010
SAUL1(ARM^7−11^)	AT1G20780	388–801	Confined patches on the PM	PMP	Drechsel et al., 2010
AtPUB43	AT1G76390	FL	PM	PMP	Vogelmann et al., 2014
AtPUB43 (ARM^7−11^)	AT1G76390	397–811	Confined patches on the PM	PMP	Vogelmann et al., 2014
FLS2	AT5G46330.1	FL	PM	IMP	Gómez-Gómez and Boller, 2000
AtFlotillin1	AT5G25250	FL	PM	PMP	Tao et al., 2019
sp-tagRFP-AFVY			Vacuolar lumen	Signal peptide (MGYMCIKISFCVMCVLGLVIVGDVAYA) from soybean PR1a precursor (Accession: NP_001238168)	Hunter et al., 2007
ACK6	AT4G28540	302–479	Cortical microtubules		Ben-Nissan et al., 2008
AtFimbrin1	AT4G26700	350–604	Actin		Wang et al., 2004
sp-tagRFP-HDEL			ER	Signal peptide (MGYMCIKISFCVMCVLGLVIVGDVAYA) from soybean PR1a precursor (Accession: NP_001238168)	Matsushima et al., 2002
**TRIFUNCTIONAL MARKERS**					
VAM7-PX-YFP-StRem1.3			PM-MVB/TP	Chimeric fusion protein	This study
Hrs-2xFYVE-YFP-StRem1.3			PM-MVB/TP	Chimeric fusion protein	This study
VAM7-PX-tagRFP-StRem1.3			PM-MVB/TP	Chimeric fusion protein	This study
Hrs-2xFYVE-tagRFP-StRem1.3			PM-MVB/TP	Chimeric fusion protein	This study
VAM7-PX-YFP-AtRem1.4			PM-MVB/TP	Chimeric fusion protein	This study
VAM7-PX^*^-YFP-AtRem1.4			PM	Chimeric fusion protein	This study
VAM7-PX-YFP^*^-StRem1.3			PM-MVB/TP	Chimeric fusion protein with mutations in the fluorophore residues of YFP: G65A, Y66A, G67A	This study

* *Mutant; ER, endoplasmic reticulum; FL, full length; IMP, integral membrane protein; MVB, multivesicular bodies; PM, plasma membrane; PM-MVB/TP, plasma membrane-multivesicular body/tonoplast tethering sites; PMP, peripheral membrane protein; SP, signal peptide; tagRFP, Tag red fluorescent protein; TP, tonoplast; YFP, yellow fluorescent protein*.

### Plant Material

Both *N. benthamiana* and *A. thaliana* plants were grown in soil (Fafard® 4M Mix). *N. benthamiana* plants were provided with a 14 h photoperiod at 25°C. Fully expanded leaves at 5 weeks were used for *A. tumefaciens* infiltrations. *A. thaliana* seeds were sown in soil and stratified at 4°C for 3 days, then the seedlings were grown in a growth chamber with a 12 h photoperiod at 20°C for 4 weeks before mesophyll protoplast isolation.

### Cloning and Construction

DNAs encoding VAM7-PX, Hrs-2xFYVE, GmPH1, FLS2, BIK1, PBS1, ACK6, and FAPP1-PH were sub-cloned from constructs as reported previously (Ben-Nissan et al., 2008; Lu et al., 2010; Meng et al., 2015; Helliwell et al., 2016). DNAs encoding AtRem1.4 (AT5G23750.1), RHA1 (AT5G45130.1), ARA7 (AT4G19640.1), ARA6 (AT3G54840.1), RABG3f (AT3G18820.1), SYT1(AT2G20990.1), AtDMP1(AT3G21520.1), AtTPK1 (AT5G55630.1), CPK21 (AT4G04720.1), AtFlotillin1 (AT5G25250), and AtFimbrin1 (At4g26700.1) were amplified from Col-0 cDNA. Genes encoding StRem1.3 (U72489.1) and Osh2p (NM_001180078) were synthesized by GenScript Corporation. The endogenous promoter *proAtRem1.4* was cloned from *A. thaliana* genomic DNA, encompassing 1.6 kb upstream from the start codon of *AtRem1.4*. All PCR amplifications were performed by High-Fidelity DNA polymerase (CloneAmp^TM^ HiFi PCR Premix, TaKaRa Bio). Primers used are listed in Supplementary Table S2. All PCR products were recombined into either pDONR207-VenusN, pDONR207-VenusC, or pDON207-xFPs which were derived from Gateway vector pDONR207, by In-Fusion® HD Cloning (TaKaRa Bio). The site-specific mutations of VAM7-PX, Hrs-2xFYVE, GmPH1, StRem1.3^*^, BIK1^*^, and FAPP1a-PH^*^ were introduced into their pDONR207 vectors using appropriate oligonucleotides in a PCR reaction to amplify the entire vector template. By using the Gateway® LR reaction (Thermo Fisher scientific Inc.), all pDONR207 vectors were subsequently transferred to a destination expression vector, either pmAEV (for cytoplasmic expression), or psAEV (providing signal peptide for ER marker), both of which were derived from the binary vector pCAMBIA (Dou et al., 2008). Both of them are driven by the Cauliflower Mosaic Virus (CaMV) 35S promoter conferring constitutive expression in plant cells. pCambia3301 was utilized to construct the endogenous-promoter expression vector pCambia3301-*ProAtRem1.4* by replacing the 35S promoter with *proAtRem1.4* using In-Fusion-mediated insertion between the *BspEI* and *NcoI* sites. All these plasmid constructs were confirmed by DNA sequencing at Center for Genome Research and Biocomputing (Oregon State University).

### Transformation in *N. benthamiana* Leaves and *A. thaliana*

For transient expression, the procedures to introduce expression vectors into *A. tumefaciens* strain GV3101, and infiltrate transformed *A. tumefaciens* into 5-week-old *N. benthamiana* leaves were carried out as described previously (Xiong et al., 2014). *A. tumefaciens* cells were infiltrated at OD_600_ of 0.1 for expression of full-length fluorescent protein tagged proteins; for co-expression of BiFC constructs, two *A. tumefaciens* cultures with OD_600_ of 0.2 each were mixed equally to reach the final OD_600_ of 0.1 each. All infiltrated *A. tumefaciens* cells were suspended in MES buffer (10 mM MgCl_2_, 10 mM MES pH 5.7, and 100 μM acetosyringone). *N. benthamiana* leaves were imaged at 3 days post-infiltration. *A. thaliana* mesophyll protoplasts were extracted from 4-week-old seedlings, and 10 μg of plasmid DNA in total was used for each transformation assay which was performed as described (Yoo et al., 2007). Protoplasts were incubated overnight in W5 buffer at 25°C before observation. Stable transgenic *A. thaliana* lines were created as described previously using floral dip procedures (Zhang et al., 2006).

### *Phytophthora* Strains and Infection Assay

A transformant of *P. capsici* strain LT263 constitutively expressing a cytosolic GFP was grown on 20% V8 vegetable juice agar plates at 25°C in the dark. Mycelia plugs with a diameter of 5 mm from the growing edge of 3~5 day old cultures were inoculated on the upper surface of freshly detached leaves which had been infiltrated with transformed *A. tumefaciens* 48 h before. The inoculated leaves were incubated at 25°C in petri dishes (150 mm diameter) containing wet paper towels to maintain 100% relative humidity. Imaging was done 24~36 h after inoculation.

### Live-Cell Imaging by Confocal Microscopy and Image Analysis

FM 4-64 (Thermo Fisher scientific Inc.) staining used a concentration of 10 μM and was performed as previously described (Günl et al., 2011). All microscopy images were obtained using a ZEISS LSM 780 NLO confocal microscope system equipped with a 458-nm argon laser for CFP (emission wavelength 560–509 nm), a 514-nm argon laser for YFP and Venus (emission wavelength 518–553 nm), and a 561 nm Diode Pumped Solid State (DPSS) laser for tagRFP and FM4-64 (emission wavelength 562–640 nm). For time-lapse imaging, movies were taken at a combined capture time of 0.97 s per frame. For 3D reconstruction, slice thickness in the *z*-axis direction of scanning was optimized at 0.6 μm with a total thickness ranging from 12–30 μm (from the top membrane layer to the middle of the cells). For tile scanning, the Plan-APO 40x/1.4 Oil DIC lens was used for imaging with the Zeiss tile-scan model combined with the Zeiss in-depth model. All microscopy images were processed using the ZEN2 (Blue edition) program.

### Quantitative Real-Time PCR

Total RNA of was extracted from ~100 mg frozen, ground leaves using the RNeasy® Mini kit (Qiagen) according to the manufacturer's directions. First strand cDNA synthesis from 2 μg total RNA was conducted using M-MuLV Reverse Transcriptase (NEB®). The cDNA was diluted to a final concentration of 5 ng/ul for qRT-PCR, then stored at −20°C until needed. The endogenous *N. benthamiana* housekeeping gene, *elongation factor 1-alpha* (*EF1*α), was used as the internal reference. Transcript levels of each gene were measured in three independent biological replicates using SYBR® *Premix Ex Taq*^TM^ kit (TaKaRa Bio) on an Applied Biosystems 7500 Fast Real-Time PCR System (Thermo Fisher scientific Inc.). The designed primers of *EF1*α and YFP used for qRT-PCR are listed in Supplementary Table S1. Data were normalized to the transcript level of *EF1a*.

## Author Contributions

KT and BT conceived and planned the experiments and wrote the manuscript. KT performed experiments and analyzed the data. JW, HW, and FA were involved in performing experiments.

### Conflict of Interest Statement

The authors declare that the research was conducted in the absence of any commercial or financial relationships that could be construed as a potential conflict of interest.

## Supplementary Material

Supplementary Figure S1 Subcellular localization of PtdIns(3)P biosensors tagged by fluorescent proteins in *N. benthamiana* leaf cortical cells. **(A)** Subcellular localization of PtdIns(3)P biosensors, VAM7-PX-YFP and Hrs-2xFYVE-YFP. The dotted boxes indicates the zoomed-in regions. **(B)** Cytoplasmic localization of mutant biosensor proteins, VAM7-PX^*^-YFP, and Hrs-2xFYVE^*^-YFP, that carry point mutations that eliminate their PtdIns(3)P binding. All scale bars represent 10 μm, except for scale bars in zoomed-in panels, that represent 5 μm.

Supplementary Figure S2 Co-localization of PtdIns(3)P biosensors with different sub-cellular marker proteins in *N. benthamiana* leaf cortical cells. **(A)** Co-expression of VAM7-PX-tagRFP or Hrs-2xFYVE-tagRFP with YFP-labeled ARA6. **(B)** Co-expression of VAM7-PX-tagRFP or Hrs-2xFYVE-tagRFP with YFP-labeled RABG3f. **(C)** Co-expression of VAM7-PX-YFP or Hrs-2xFYVE-YFP with tagRFP-labeled tonoplast marker AtTPK1. **(D)** Subcellular localization of PM-associated remorin StRem1.3 fused with YFP. **(E)** Localization of VAM7-PX-YFP or Hrs-2xFYVE-YFP relative to co-expressed tagRFP-StRem1.3. Right panel: fluorescence intensity plot along a transect shown by the white line in the merged image. Scale bars in all panels represent 10 μm.

Supplementary Figure S3 BiFC complexes containing PtdIns(3)P biosensors and StRem1.3 produce large patches on the PM of *A. thaliana* mesophyll protoplasts. VenusC–StRem1.3 was transiently co-expressed with PtdIns(3)P biosensors VAM7-PX-VenusN or Hrs-2xFYVE-VenusN. Scale is identical in all panels and represents 10 μm.

Supplementary Figure S4 Subcellular localization of trifunctional fusion proteins expressed at different levels in *N. benthamiana* leaf cortical cells. **(A)** Trifunctional fusion proteins contained a PtdIns(3)P biosensor (VAM7-PX or Hrs-2xFYVE), fused to YFP or tagRFP and also to StRem1.3, in that order from N-terminus to C-terminus. Expression of these fusion proteins in *N. benthamiana* cells produced membrane patches without the use of BiFC. The membrane patches in bigger sizes are highlighted by the solid circles, and vice versa for the dotted circles. **(B)** Expression level determines the sizes of patches produced by trifunctional fusion protein VAM7-PX-YFP-AtRem1.4. Expression was driven by either the native promoter *ProAtRem1.4* or the highly active *CaMV35S* promoter. Dashed boxes indicate regions enlarged in the top layer views shown in the center panels. Mutations in the PtdIns(3)P binding site of VAM7-PX^*^-YFP-AtRem1.4 abolishes the formation of patches with either promoter. **(C)** Subcellular localization of AtRem1.4 fused with YFP. **(D)** Quantitative Real-Time PCR assay of the transcript levels of fusion constructs in **(B)** above. The experiments were replicated three times independently. All data were normalized relative to the transcript level of the internal reference gene *EF1a*. Error bars represent s.e. **(E)** Fluorescence distribution of VAM7-PX-YFP-AtRem1.4 and VAM7-PX^*^-YFP-AtRem1.4 produced by expression driven by the native promoter *ProAtRem1.4* in transgenic *Arabidopsis* lines. The scale bars in **(A–E)** represent 10 μm.

Supplementary Figure S5 Subcellular localizations of wildtype and mutant plasma membrane proteins including PtdIns(4)P biosensors in *N. benthamiana* leaf cortical cells. **(A)** Localization of YFP-fused peripheral membrane proteins, BIK1, PBS1, and CPK21, and PtdIns(4)P biosensors, FAPP1a and Osh2p. **(B)** Localization of YFP-fused mutant versions of PM proteins and PtdIns(4)P biosensors (StRem1.3^*^, BIK1^*^, and FAPP1a^*^) carrying mutations in residues required for binding to the PM or to PtdIns(4)P respectively. **(C)** Localization of BiFC complexes formed by co-expression of PM-non-binding mutants VenusN-StRem1.3^*^, BIK1^*^-VenusN or FAPP1a-PH^*^-VenusN with either VenusC-VAM7-PX or VenusC-Hrs-2xFYVE. **(D)** Localization of BiFC complexes formed by co-expression of VenusN-fused PtdIns(4)P-binding protein Osh2p together with VenusC-fused VAM7-PX or Hrs-2xFYVE. The scale bar represents 10 μm in all panels.

Supplementary Figure S6 Subcellular localizations of proteins associated with MVBs and the tonoplast in *N. benthamiana* leaf cortical cells. Rab5-type GTPases RHA1, ARA7, and ARA6, and Rab7-type GTPase RABG3f were fused with YFP. Dashed boxes indicate regions enlarged in bottom panels with the scale bar representing 5 μm. All scale bars in other panels represent 10 μm.

Supplementary Figure S7 Confocal imaging of PM-tethering with either the tonoplast or MVBs, and the subcellular localizations of tonoplast-associated proteins in *N. benthamiana* leaf cortical cells. **(A)** Distinct tonoplast- and MVB-associated patches revealed by co-expression of YFP-labeled RHA1 or ARA7 with Hrs-2xFYVE-tagRFP-StRem1.3. Examples of tonoplast- and MVB-associated patches are highlighted with open and filled arrows, respectively. Punctae associating with tonoplast or MVBs are highlighted by dotted and solid arrows, respectively. **(B)** Localization of GFP-fused tonoplast-associated proteins, AtDMP1, and AtTPK1. All scale bars represent 10 μm.

Supplementary Figure S8 Exclusion of PM proteins by membrane patches in *N. benthamiana* leaf cortical cells is not an artifact of confocal microscopic image analysis. **(A)** Non-fluorescent membrane patches were produced by expression of colorless mutant fusion protein VAM7-PX-YFP^*^-StRem1.3. These patches excluded PM-localized AtFlotillin-YFP and FLS2-YFP, whereas AtRem1.4, the closest *Arabidopsis* homolog of StRem1.3 was enriched in the colorless patches. **(B)** The presence of fluorescent membrane patches produced by Hrs-2xFYVE-YFP-StRem1.3 did not affect coincident visualization of vacuolar lumen marker SP-tagRFP-AFVY. All scale bars represent 10 μm.

Supplementary Figure S9 Relationship of membrane patches with the cytoskeleton and endoplasmic reticulum in *N. benthamiana* leaf cortical cells. **(A)** Relationship of membrane patches produced by Hrs-2xFYVE-YFP-StRem1.3 to the cortical microtubules labeled with tagRFP fused to *Arabidopsis* Casein Kinase 1-Like 6 (ACK6). Enlarged panels show examples of patches being divided by microtubules (indicated by empty arrows). The white scale bars represent 5 μm. **(B)** Maximum intensity and orthogonal projections of cells exhibiting membrane patches produced by Hrs-2xFYVE-YFP-StRem1.3 and co-expressing the actin filament marker AtFimbrin1-tagRFP. **(C)** Relationship of membrane patches produced by Hrs-2xFYVE-YFP-StRem1.3 to the endoplasmic reticulum tagged by ER lumenal marker SP-tagRFP-HDEL. All other scale bars represent 10 μm.

Supplementary Figure S10 Subcellular localization assay of SAUL1 paralog AtPUB43 in *N. benthamiana* leaf cortical cells. **(A)** Subcellular localization of YFP-tagged full length AtPUB43, and YFP-tagged AtPUB43 C-terminal ARM repeats 7–11. **(B)** Full-length AtPUB43 co-expressed with either ARA6, or VAM7-PX, or Hrs-2xFYVE in BiFC complexes form patches indicative of PM-MVB/TP tethering. **(C)** Co-localization of membrane patches produced by expression of Hrs-2xFYVE-YFP-StRem1.3 with patches produced by tagRFP-AtPUB43(ARM^7−11^). **(D)** Co-localization of membrane patches produced by expression of YFP-SAUL1(ARM^7−11^) and tagRFP-AtPUB43(ARM^7−11^). All scale bars represent 10 μm.

Supplementary Figure S11 Distribution of fluorescent tonoplast and MVB marker proteins in the presence of membrane patches created by expression of tagRFP-AtPUB43(ARM^7−11^) in *N. benthamiana* leaf cortical cells. **(A)** Distinct tonoplast- and MVB-associated patches revealed by co-expression of YFP- or GFP-fused Hrs-2xFYVE, ARA6, AtDMP1 or AtTPK1 together with tagRFP-AtPUB43(ARM^7−11^). Examples of tonoplast- and MVB-associated patches are highlighted with open and filled arrows, respectively. Punctae associating with tonoplast or MVBs are highlighted by dotted or solid arrows, respectively. **(B)** Distribution of cytoplasmic GFP co-expressed with tagRFP-AtPUB43(ARM^7−11^). Examples of tonoplast-associated patches from which GFP has been excluded are highlighted with open arrows while MVB-associated patches from which GFP has not been fully excluded are highlighted with filled arrows. All scale bars represent 10 μm.

Supplementary Figure S12 Distribution of the fluorescently tagged full-length AtPUB43 in *N. benthamiana* leaf cortical cells during pathogen infection. **(A)** Cortical cells expressing YFP-tagged AtPUB43 were infected by GFP-tagged oomycete *P. capsici* (artificially represented by the red color). Top panel: tile-scan imaging showing the changing distribution of YFP-AtPUB43 on the PM associated with the progress of *P. capsici* infection. Scale bar in this panel represents 50 μm. Lower panel: details of the distribution of YFP-AtPUB43 in necrotic (outlined by gray box), infected (outlined by purple box) and uninfected cells (outlined by blue box). **(B)** Distribution of ARA6-YFP compared to YFP-AtPUB43 in uninfected and infected cells. The tonoplast- and MVB-associated patches formed by YFP- AtPUB43 were revealed by ARA6-YFP as highlighted with open and filled arrows, respectively. Likewise, punctae associating with the tonoplast or MVBs, are highlighted by dotted and solid arrows, respectively. All other scale bars represent 10 μm.

Supplementary Movie S1 Dynamic tubes and vesicles labeled by the AtDMP1-GFP interacting over time with membrane patches produced by Hrs-2xFYVE-tagRFP-StRem1.3. Related to Figure 3B.

Supplementary Movie S2 Video of z-axis scan of cells co-expressing soluble tagRFP and AtDMP1-GFP. Related to the left panel of Figure 4B.

Supplementary Movie S3 Video of z-axis scan of cells co-expressing soluble tagRFP and AtDMP1-GFP together with the colorless fusion protein VAM7-PX-YFP^*^-StRem1.3. Related to the right panel of Figure 4B.

Supplementary Table S1 Amino acid sequences, plasmid constructs for expression, and other details of protein fusions used in this study.

Supplementary Table S2 Primer information.
