# Supplementary figures and images for: Tethering of Multi-Vesicular Bodies and the Tonoplast to the Plasma Membrane in Plants
Source: Front Plant Sci. 2019 May 22;10:636. doi: 10.3389/fpls.2019.00636 (PMC6662526; doi:10.3389/fpls.2019.00636)

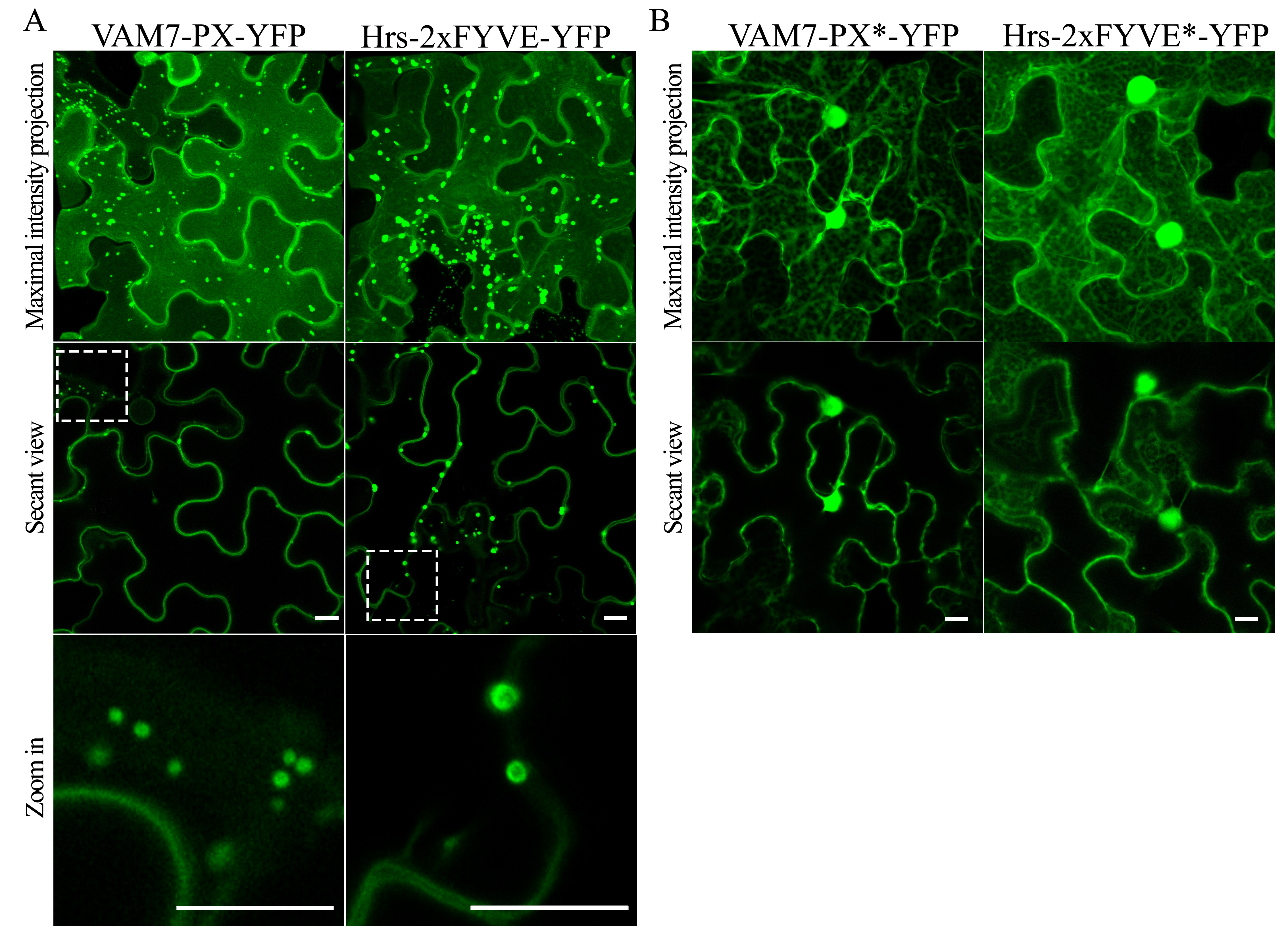

Supplement: Supplementary Figure S1 — Subcellular localization of PtdIns(3)P biosensors tagged by fluorescent proteins in N. benthamiana leaf cortical cells. (A) Subcellular localization of PtdIns(3)P biosensors, VAM7-PX-YFP and Hrs-2xFYVE-YFP. The dotted boxes indicates the zoomed-in regions. (B) Cytoplasmic localization of mutant biosensor proteins, VAM7-PX*-YFP, and Hrs-2xFYVE*-YFP, that carry point mutations that eliminate their PtdIns(3)P binding. All scale bars represent 10 μm, except for scale bars in zoomed-in panels, that represent 5 μm. [file Image_1.JPEG]

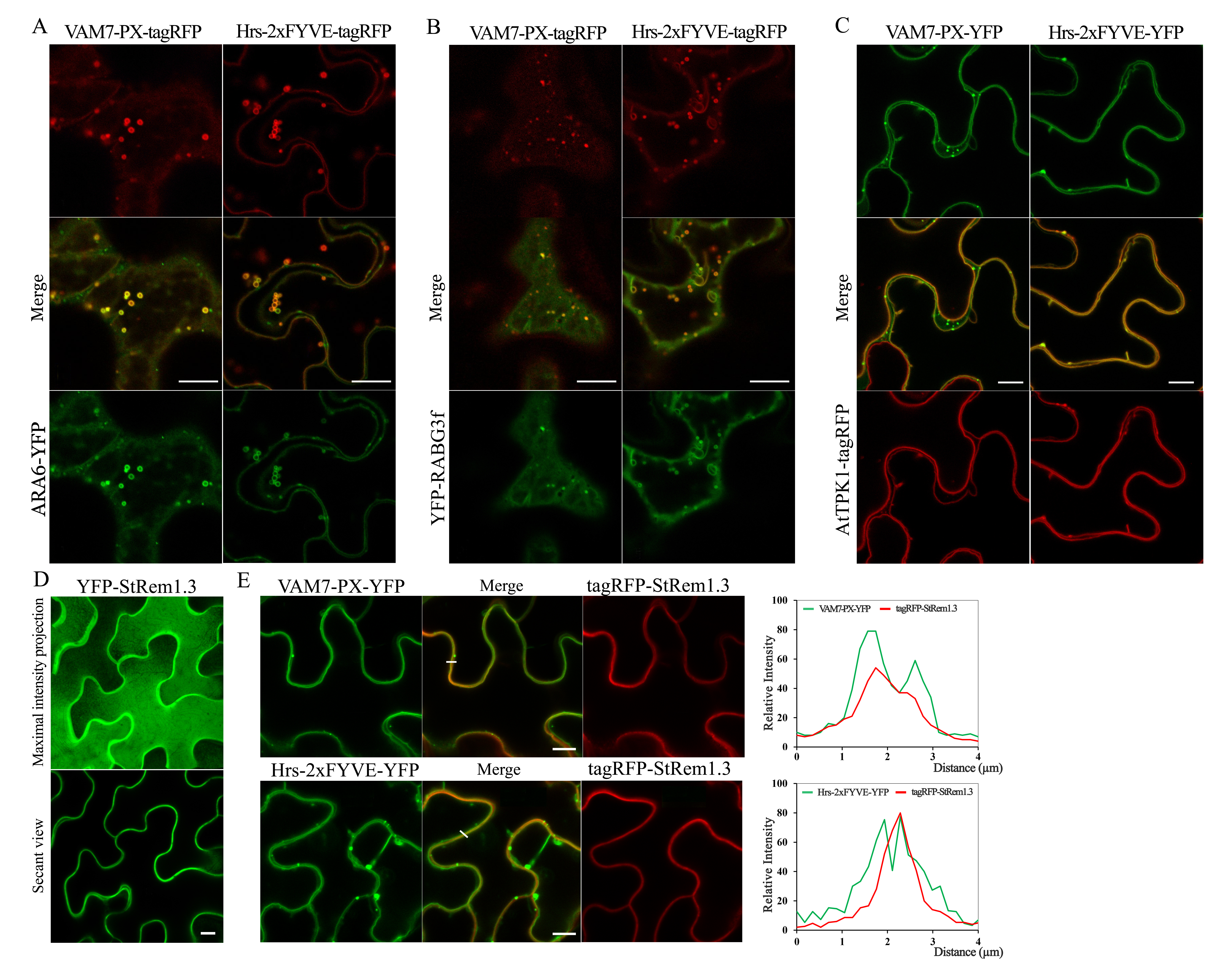

Supplement: Supplementary Figure S2 — Co-localization of PtdIns(3)P biosensors with different sub-cellular marker proteins in N. benthamiana leaf cortical cells. (A) Co-expression of VAM7-PX-tagRFP or Hrs-2xFYVE-tagRFP with YFP-labeled ARA6. (B) Co-expression of VAM7-PX-tagRFP or Hrs-2xFYVE-tagRFP with YFP-labeled RABG3f. (C) Co-expression of VAM7-PX-YFP or Hrs-2xFYVE-YFP with tagRFP-labeled tonoplast marker AtTPK1. (D) Subcellular localization of PM-associated remorin StRem1.3 fused with YFP. (E) Localization of VAM7-PX-YFP or Hrs-2xFYVE-YFP relative to co-expressed tagRFP-StRem1.3. Right panel: fluorescence intensity plot along a transect shown by the white line in the merged image. Scale bars in all panels represent 10 μm. [file Image_2.JPEG]

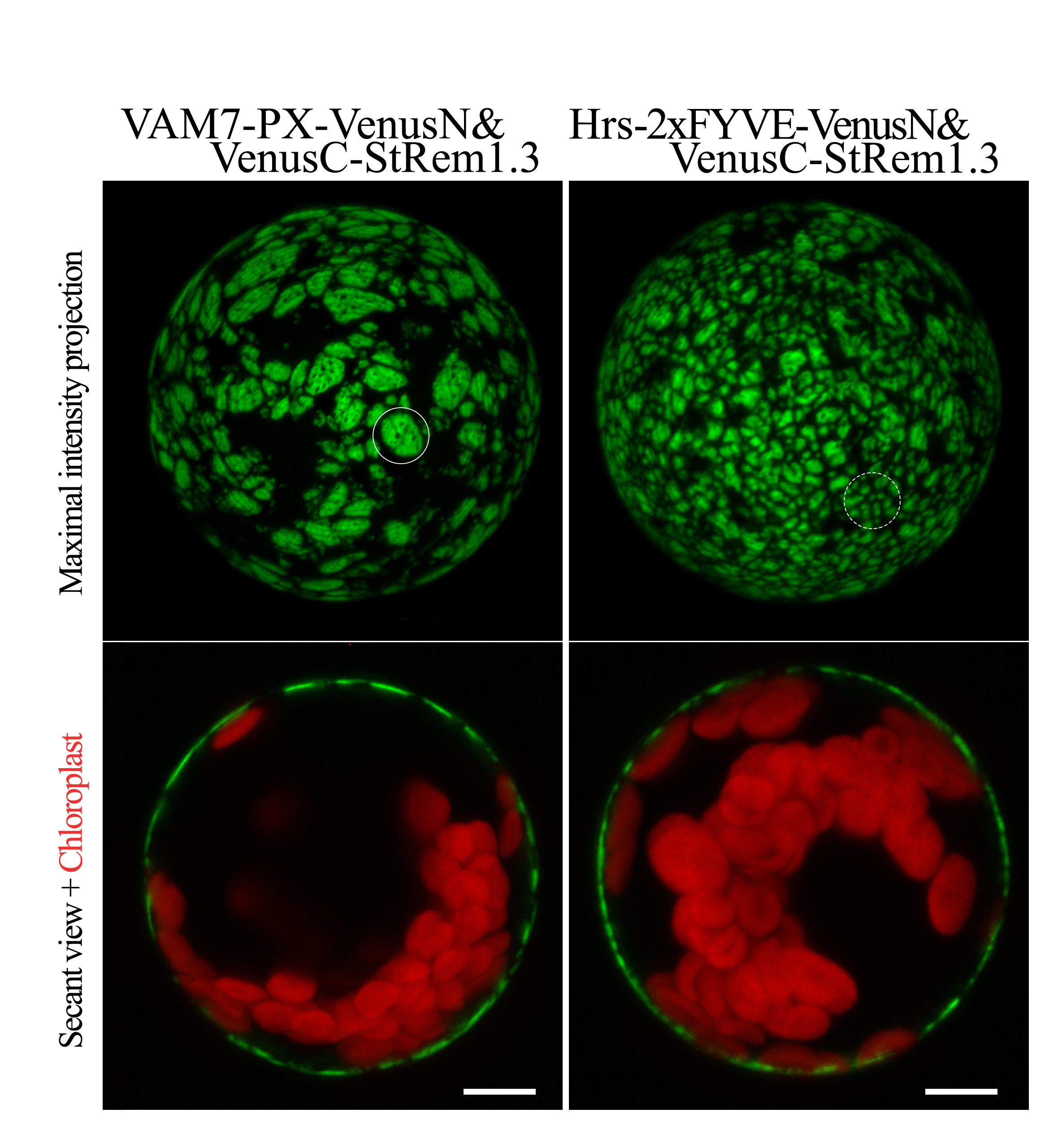

Supplement: Supplementary Figure S3 — BiFC complexes containing PtdIns(3)P biosensors and StRem1.3 produce large patches on the PM of A. thaliana mesophyll protoplasts. VenusC–StRem1.3 was transiently co-expressed with PtdIns(3)P biosensors VAM7-PX-VenusN or Hrs-2xFYVE-VenusN. Scale is identical in all panels and represents 10 μm. [file Image_3.JPEG]

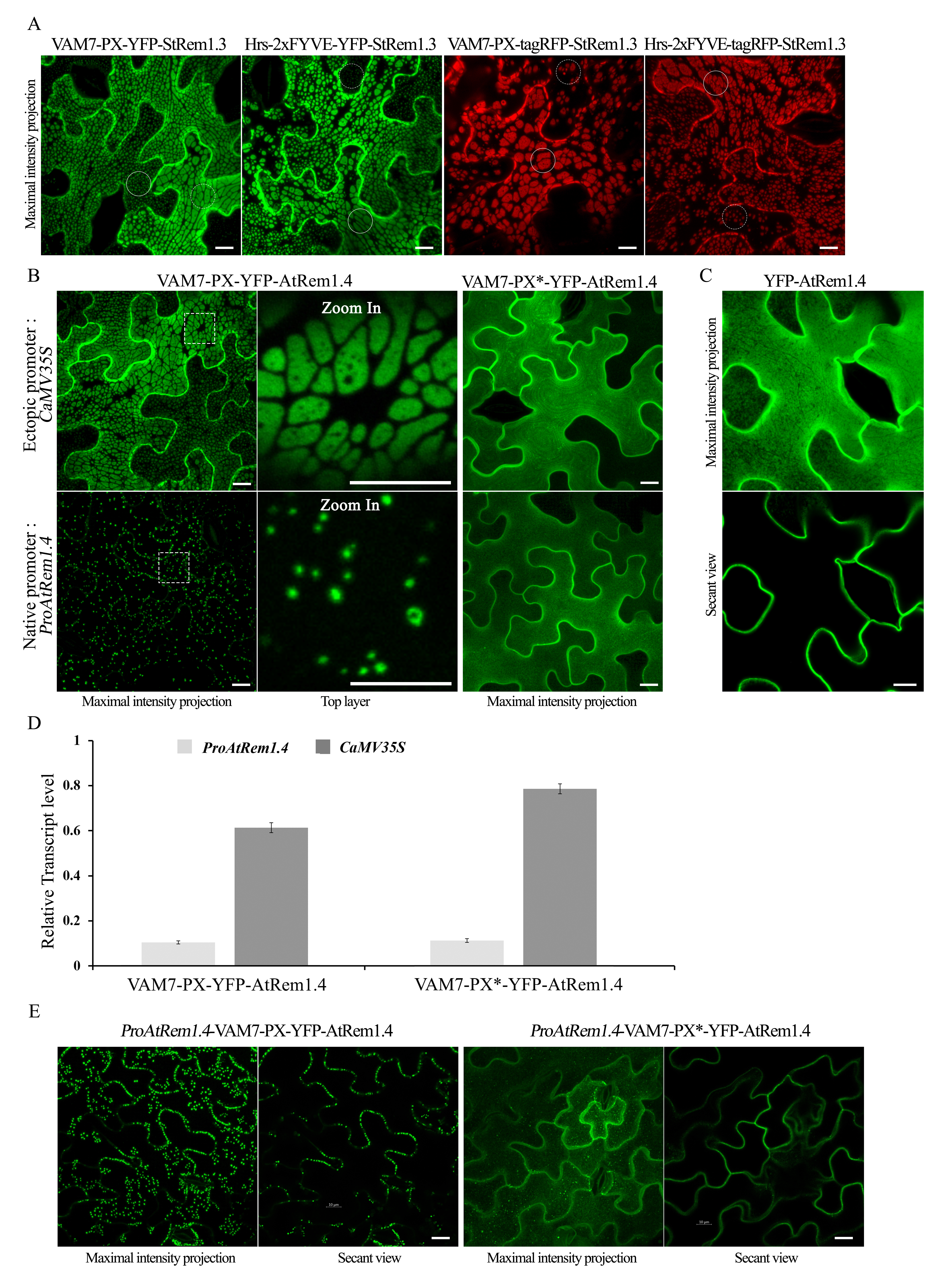

Supplement: Supplementary Figure S4 — Subcellular localization of trifunctional fusion proteins expressed at different levels in N. benthamiana leaf cortical cells. (A) Trifunctional fusion proteins contained a PtdIns(3)P biosensor (VAM7-PX or Hrs-2xFYVE), fused to YFP or tagRFP and also to StRem1.3, in that order from N-terminus to C-terminus. Expression of these fusion proteins in N. benthamiana cells produced membrane patches without the use of BiFC. The membrane patches in bigger sizes are highlighted by the solid circles, and vice versa for the dotted circles. (B) Expression level determines the sizes of patches produced by trifunctional fusion protein VAM7-PX-YFP-AtRem1.4. Expression was driven by either the native promoter ProAtRem1.4 or the highly active CaMV35S promoter. Dashed boxes indicate regions enlarged in the top layer views shown in the center panels. Mutations in the PtdIns(3)P binding site of VAM7-PX*-YFP-AtRem1.4 abolishes the formation of patches with either promoter. (C) Subcellular localization of AtRem1.4 fused with YFP. (D) Quantitative Real-Time PCR assay of the transcript levels of fusion constructs in (B) above. The experiments were replicated three times independently. All data were normalized relative to the transcript level of the internal reference gene EF1a. Error bars represent s.e. (E) Fluorescence distribution of VAM7-PX-YFP-AtRem1.4 and VAM7-PX*-YFP-AtRem1.4 produced by expression driven by the native promoter ProAtRem1.4 in transgenic Arabidopsis lines. The scale bars in (A–E) represent 10 μm. [file Image_4.JPEG]

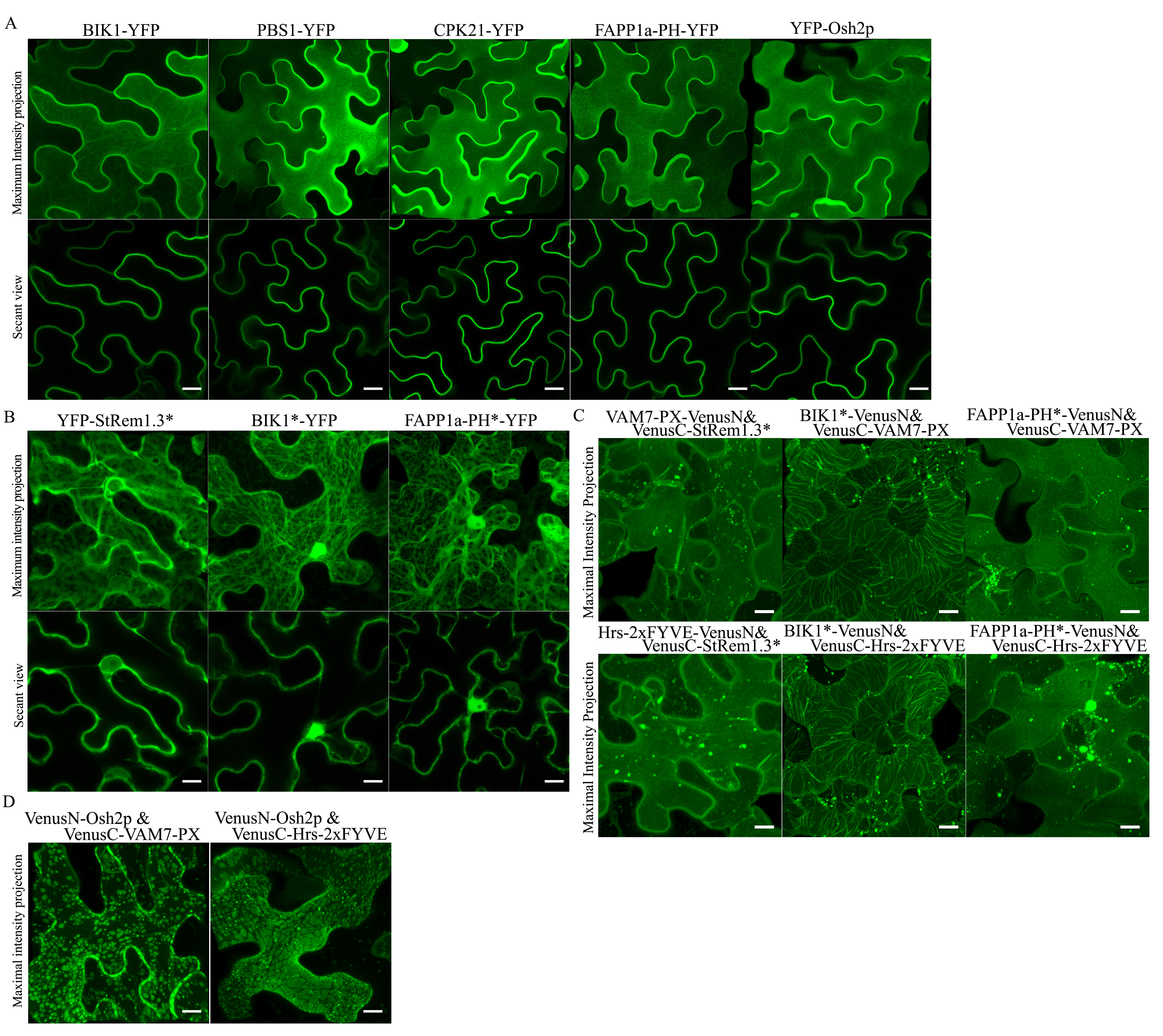

Supplement: Supplementary Figure S5 — Subcellular localizations of wildtype and mutant plasma membrane proteins including PtdIns(4)P biosensors in N. benthamiana leaf cortical cells. (A) Localization of YFP-fused peripheral membrane proteins, BIK1, PBS1, and CPK21, and PtdIns(4)P biosensors, FAPP1a and Osh2p. (B) Localization of YFP-fused mutant versions of PM proteins and PtdIns(4)P biosensors (StRem1.3*, BIK1*, and FAPP1a*) carrying mutations in residues required for binding to the PM or to PtdIns(4)P respectively. (C) Localization of BiFC complexes formed by co-expression of PM-non-binding mutants VenusN-StRem1.3*, BIK1*-VenusN or FAPP1a-PH*-VenusN with either VenusC-VAM7-PX or VenusC-Hrs-2xFYVE. (D) Localization of BiFC complexes formed by co-expression of VenusN-fused PtdIns(4)P-binding protein Osh2p together with VenusC-fused VAM7-PX or Hrs-2xFYVE. The scale bar represents 10 μm in all panels. [file Image_5.JPEG]

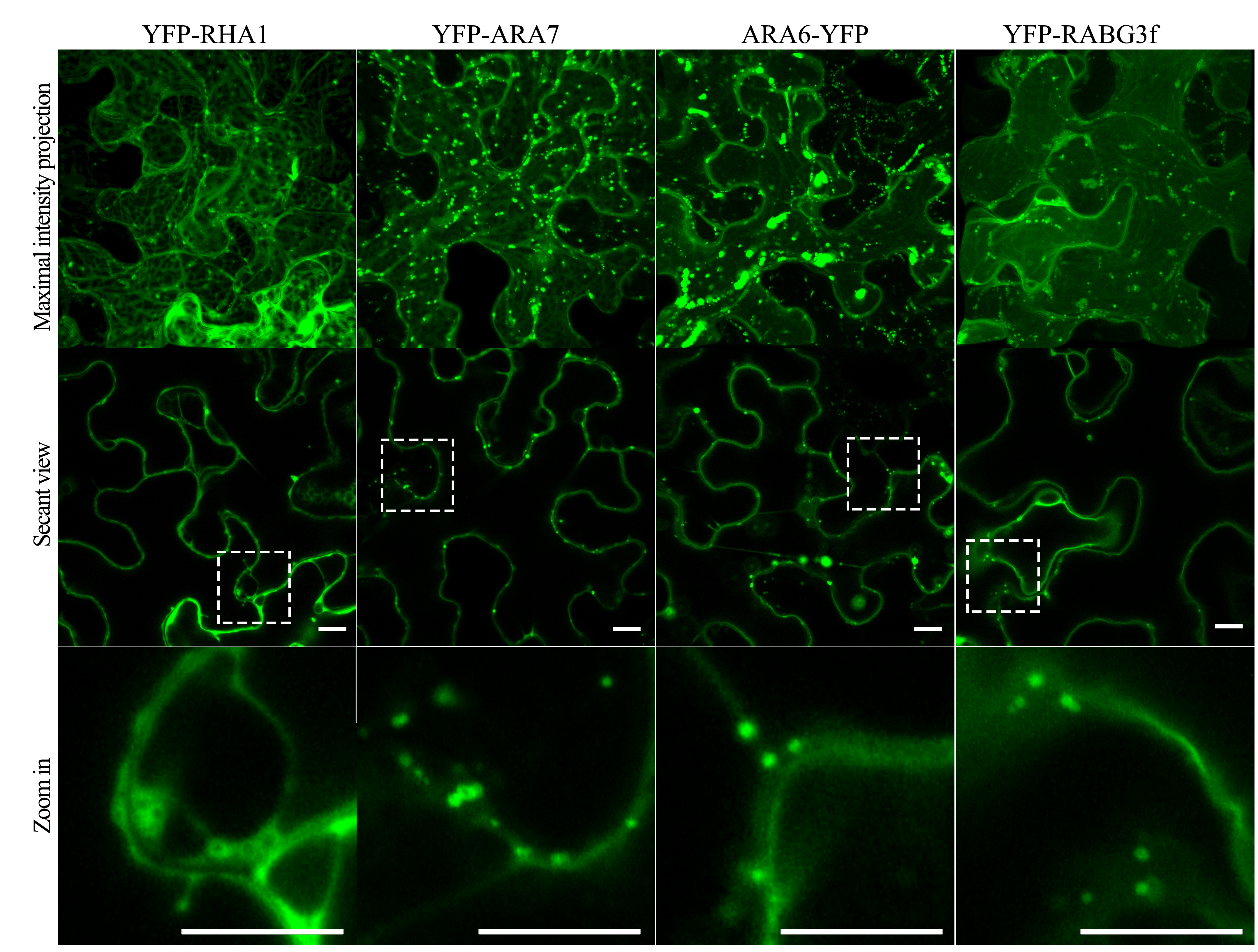

Supplement: Supplementary Figure S6 — Subcellular localizations of proteins associated with MVBs and the tonoplast in N. benthamiana leaf cortical cells. Rab5-type GTPases RHA1, ARA7, and ARA6, and Rab7-type GTPase RABG3f were fused with YFP. Dashed boxes indicate regions enlarged in bottom panels with the scale bar representing 5 μm. All scale bars in other panels represent 10 μm. [file Image_6.JPEG]

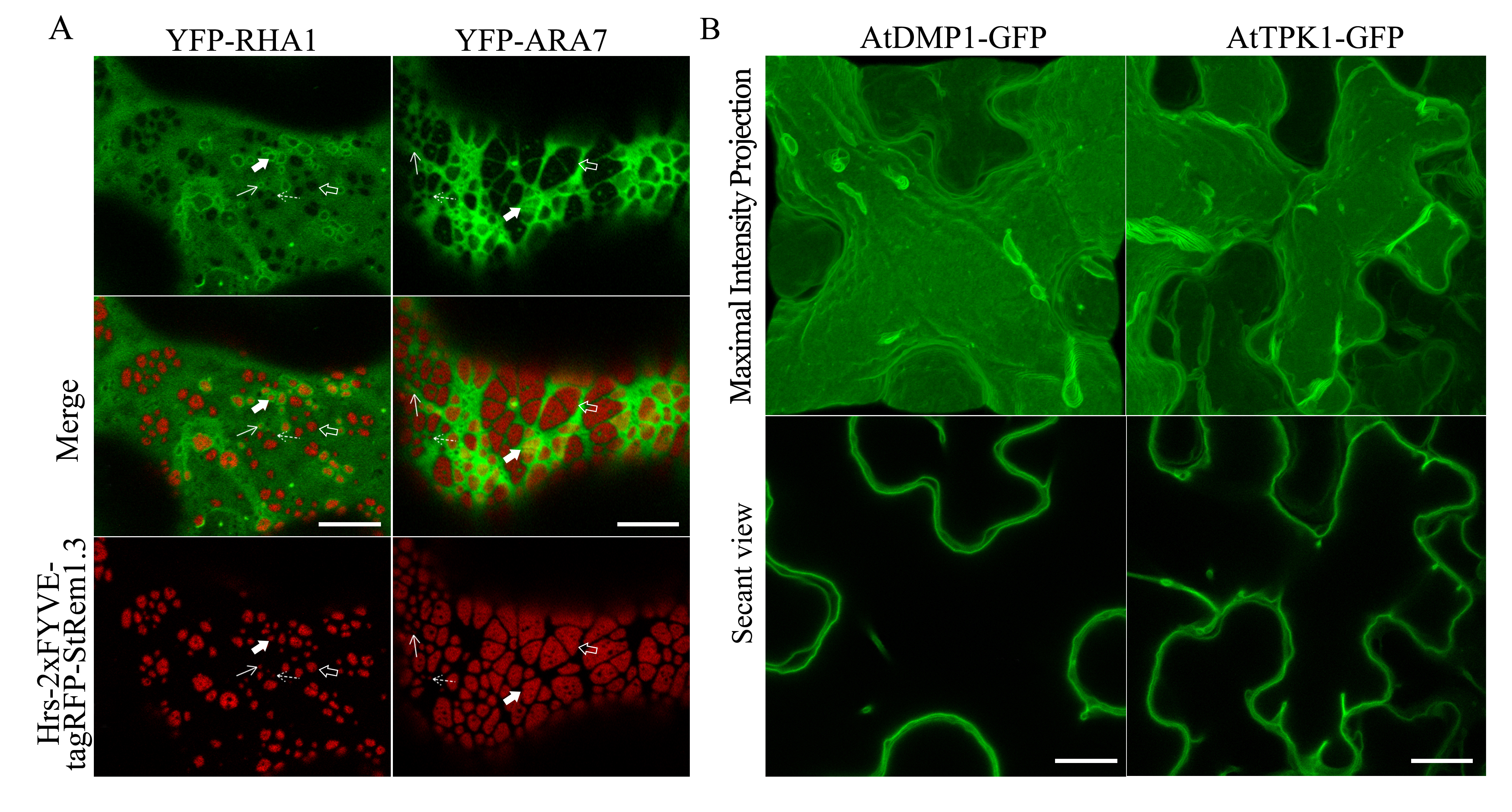

Supplement: Supplementary Figure S7 — Confocal imaging of PM-tethering with either the tonoplast or MVBs, and the subcellular localizations of tonoplast-associated proteins in N. benthamiana leaf cortical cells. (A) Distinct tonoplast- and MVB-associated patches revealed by co-expression of YFP-labeled RHA1 or ARA7 with Hrs-2xFYVE-tagRFP-StRem1.3. Examples of tonoplast- and MVB-associated patches are highlighted with open and filled arrows, respectively. Punctae associating with tonoplast or MVBs are highlighted by dotted and solid arrows, respectively. (B) Localization of GFP-fused tonoplast-associated proteins, AtDMP1, and AtTPK1. All scale bars represent 10 μm. [file Image_7.JPEG]

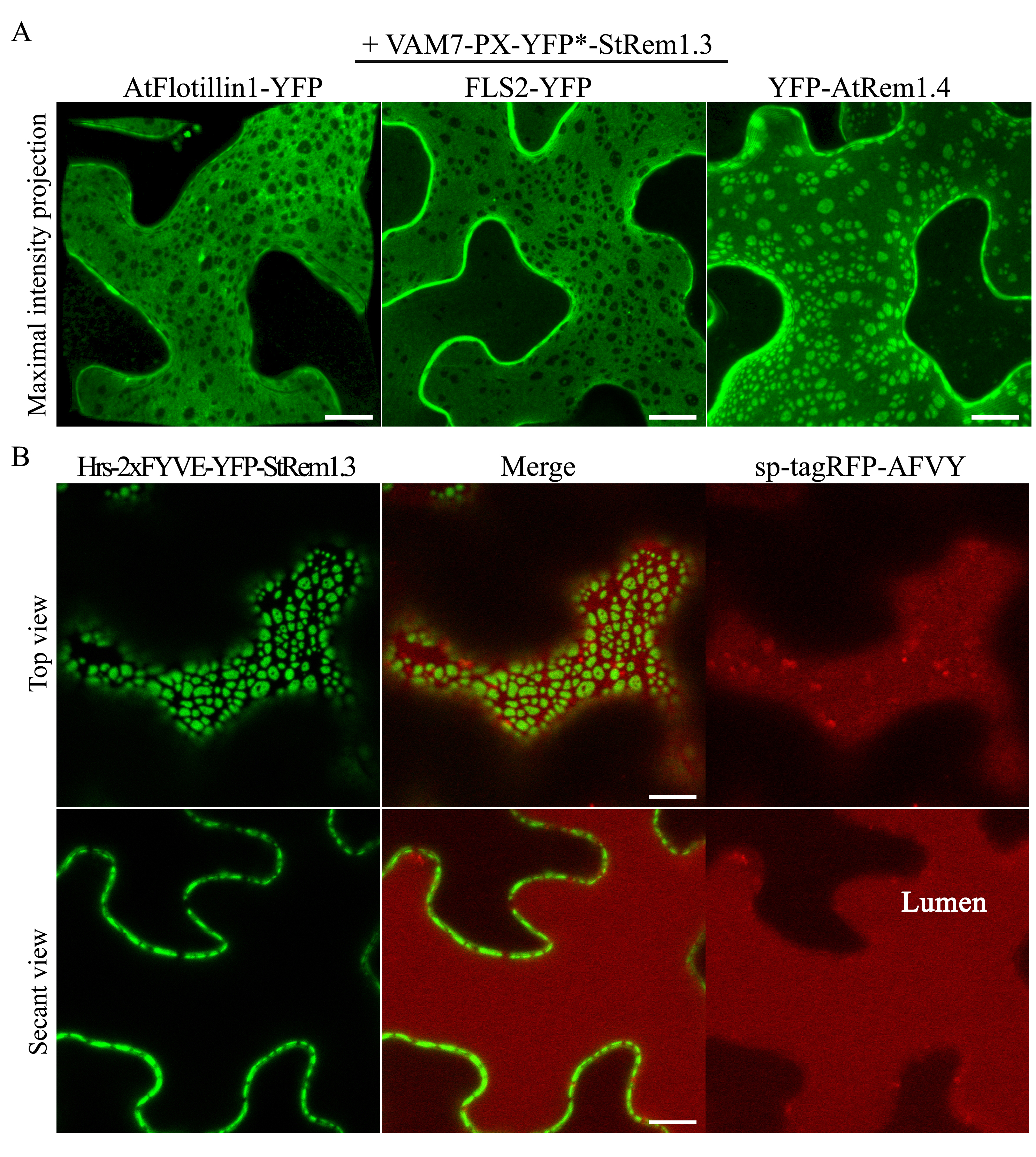

Supplement: Supplementary Figure S8 — Exclusion of PM proteins by membrane patches in N. benthamiana leaf cortical cells is not an artifact of confocal microscopic image analysis. (A) Non-fluorescent membrane patches were produced by expression of colorless mutant fusion protein VAM7-PX-YFP*-StRem1.3. These patches excluded PM-localized AtFlotillin-YFP and FLS2-YFP, whereas AtRem1.4, the closest Arabidopsis homolog of StRem1.3 was enriched in the colorless patches. (B) The presence of fluorescent membrane patches produced by Hrs-2xFYVE-YFP-StRem1.3 did not affect coincident visualization of vacuolar lumen marker SP-tagRFP-AFVY. All scale bars represent 10 μm. [file Image_8.JPEG]

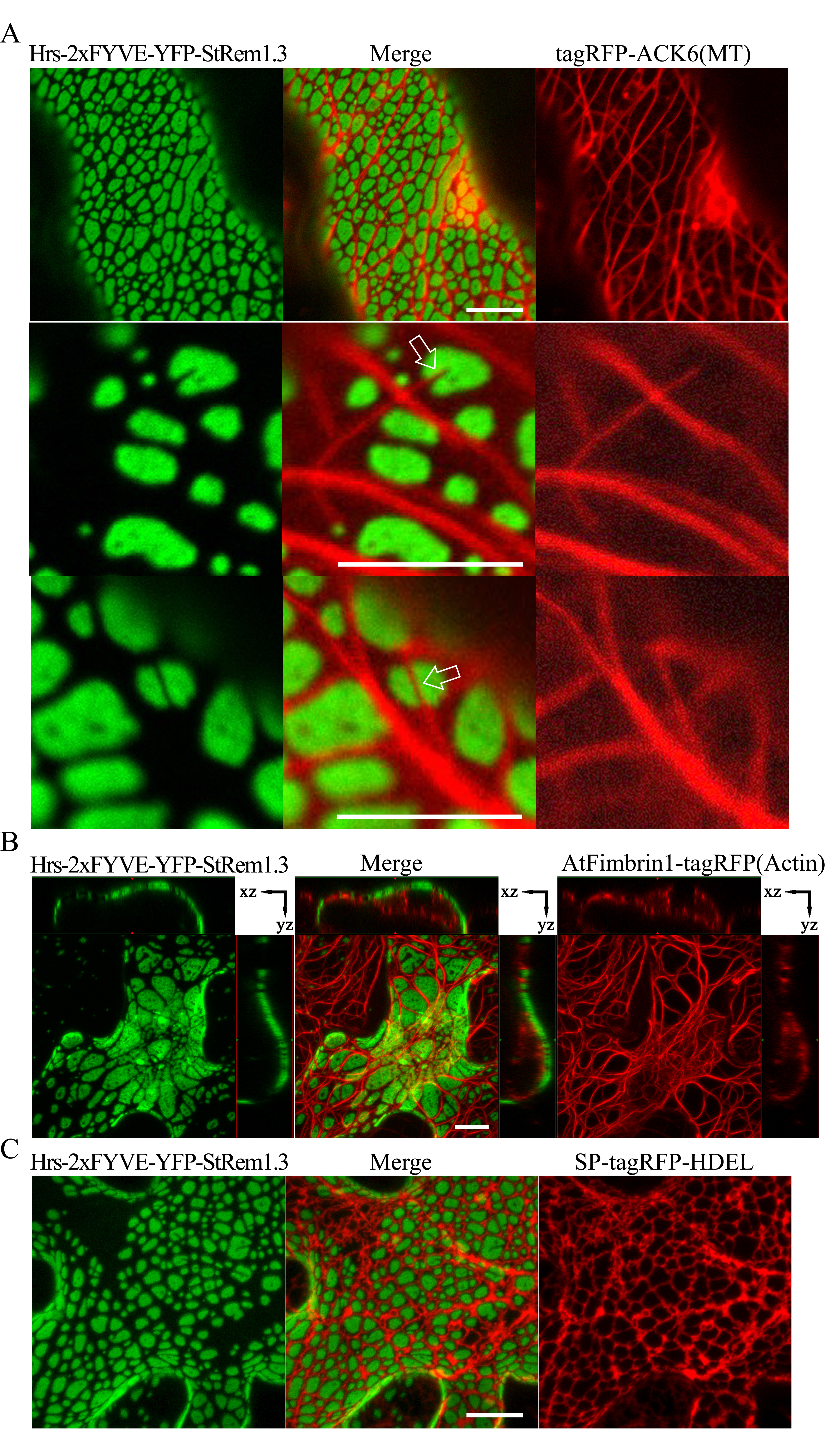

Supplement: Supplementary Figure S9 — Relationship of membrane patches with the cytoskeleton and endoplasmic reticulum in N. benthamiana leaf cortical cells. (A) Relationship of membrane patches produced by Hrs-2xFYVE-YFP-StRem1.3 to the cortical microtubules labeled with tagRFP fused to Arabidopsis Casein Kinase 1-Like 6 (ACK6). Enlarged panels show examples of patches being divided by microtubules (indicated by empty arrows). The white scale bars represent 5 μm. (B) Maximum intensity and orthogonal projections of cells exhibiting membrane patches produced by Hrs-2xFYVE-YFP-StRem1.3 and co-expressing the actin filament marker AtFimbrin1-tagRFP. (C) Relationship of membrane patches produced by Hrs-2xFYVE-YFP-StRem1.3 to the endoplasmic reticulum tagged by ER lumenal marker SP-tagRFP-HDEL. All other scale bars represent 10 μm. [file Image_9.JPEG]

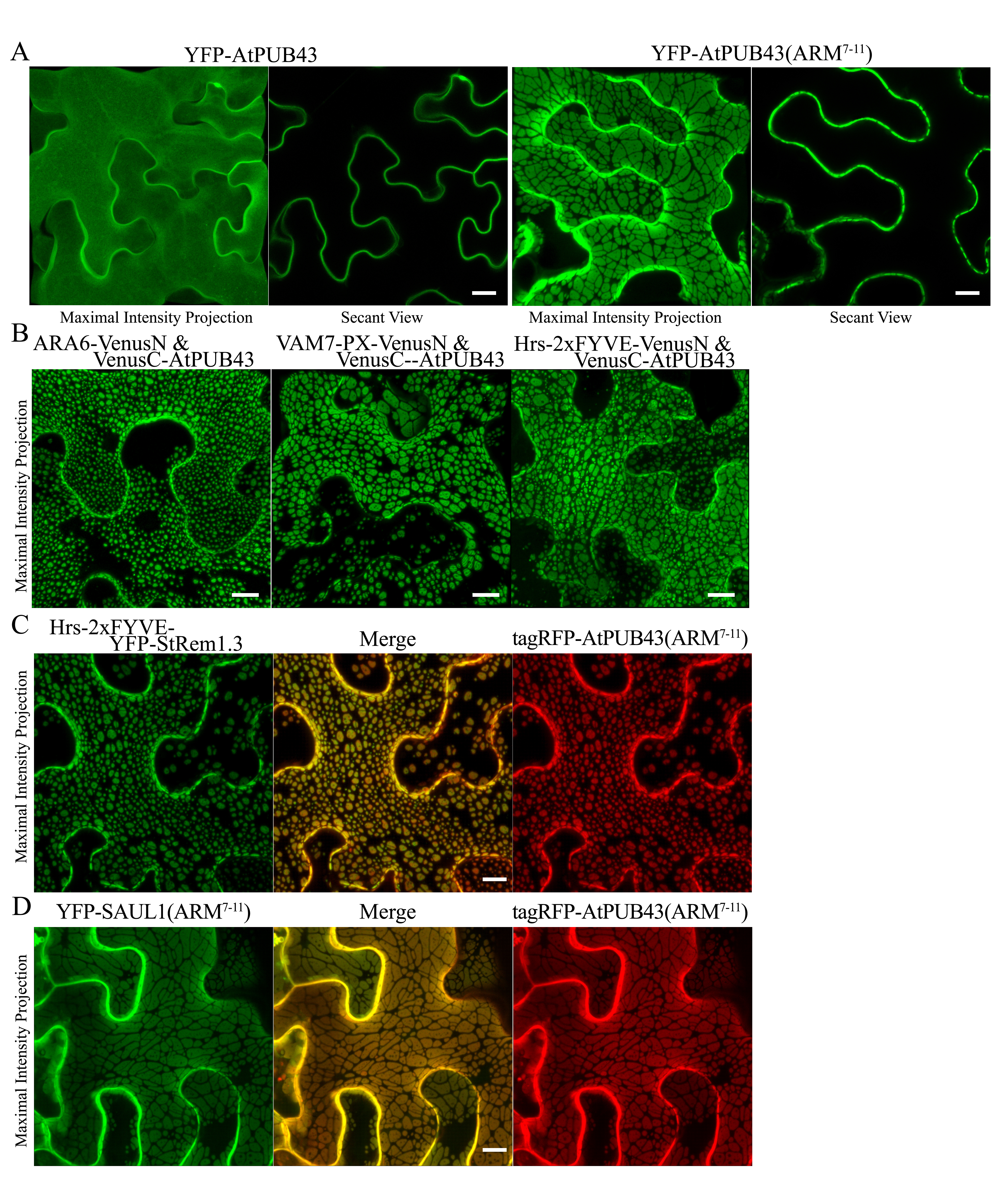

Supplement: Supplementary Figure S10 — Subcellular localization assay of SAUL1 paralog AtPUB43 in N. benthamiana leaf cortical cells. (A) Subcellular localization of YFP-tagged full length AtPUB43, and YFP-tagged AtPUB43 C-terminal ARM repeats 7–11. (B) Full-length AtPUB43 co-expressed with either ARA6, or VAM7-PX, or Hrs-2xFYVE in BiFC complexes form patches indicative of PM-MVB/TP tethering. (C) Co-localization of membrane patches produced by expression of Hrs-2xFYVE-YFP-StRem1.3 with patches produced by tagRFP-AtPUB43(ARM7−11). (D) Co-localization of membrane patches produced by expression of YFP-SAUL1(ARM7−11) and tagRFP-AtPUB43(ARM7−11). All scale bars represent 10 μm. [file Image_10.JPEG]

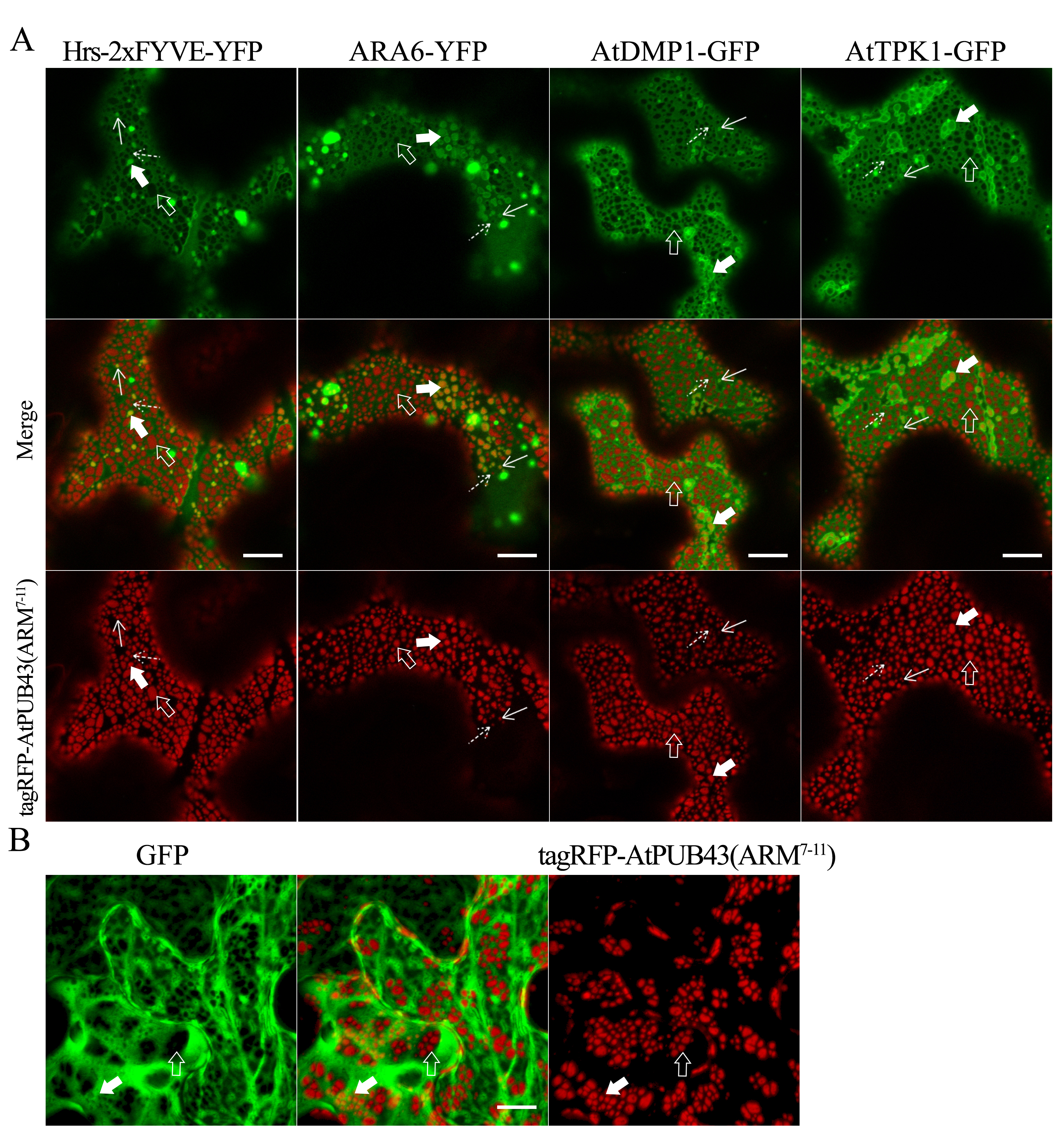

Supplement: Supplementary Figure S11 — Distribution of fluorescent tonoplast and MVB marker proteins in the presence of membrane patches created by expression of tagRFP-AtPUB43(ARM7−11) in N. benthamiana leaf cortical cells. (A) Distinct tonoplast- and MVB-associated patches revealed by co-expression of YFP- or GFP-fused Hrs-2xFYVE, ARA6, AtDMP1 or AtTPK1 together with tagRFP-AtPUB43(ARM7−11). Examples of tonoplast- and MVB-associated patches are highlighted with open and filled arrows, respectively. Punctae associating with tonoplast or MVBs are highlighted by dotted or solid arrows, respectively. (B) Distribution of cytoplasmic GFP co-expressed with tagRFP-AtPUB43(ARM7−11). Examples of tonoplast-associated patches from which GFP has been excluded are highlighted with open arrows while MVB-associated patches from which GFP has not been fully excluded are highlighted with filled arrows. All scale bars represent 10 μm. [file Image_11.JPEG]

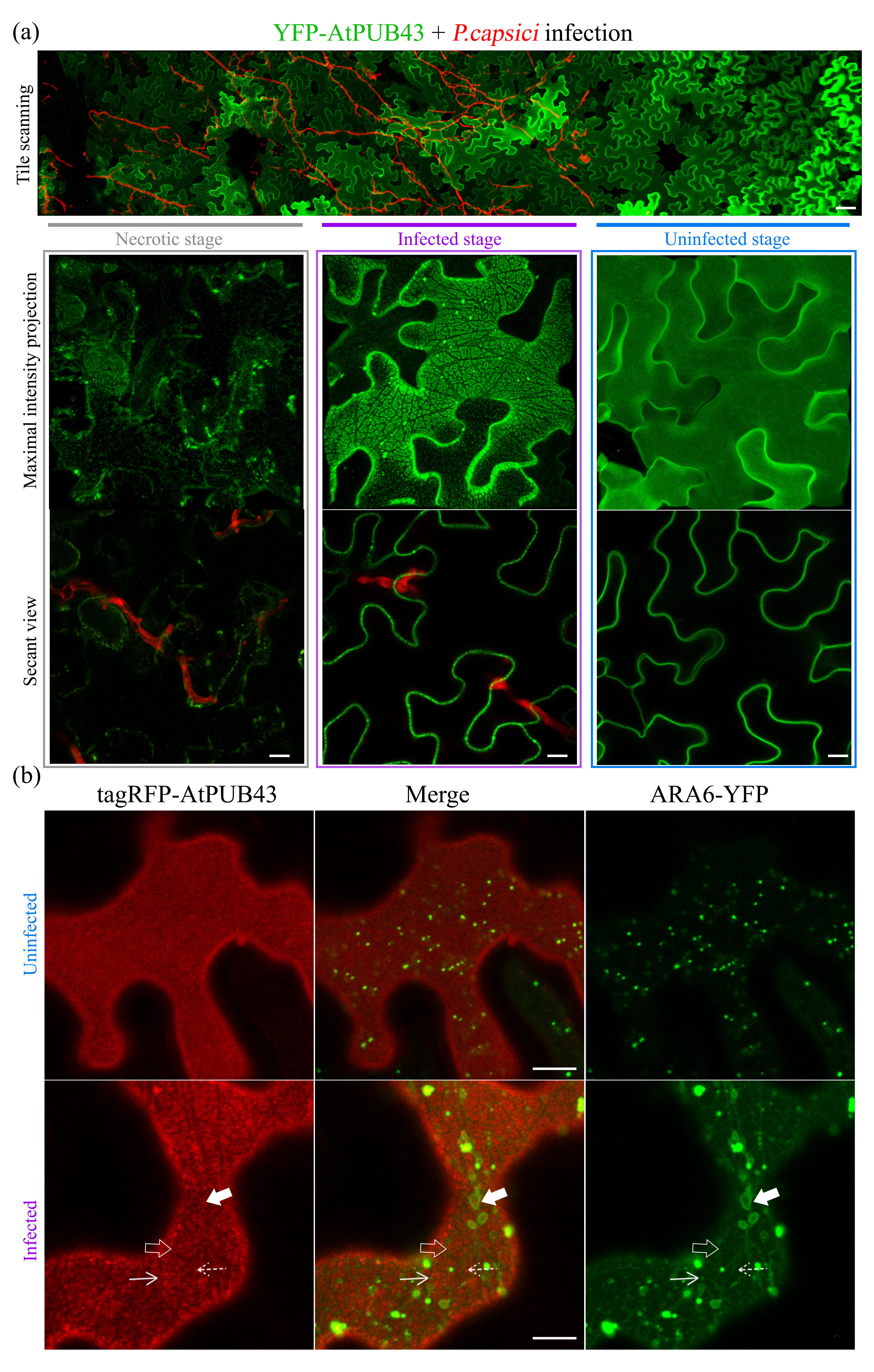

Supplement: Supplementary Figure S12 — Distribution of the fluorescently tagged full-length AtPUB43 in N. benthamiana leaf cortical cells during pathogen infection. (A) Cortical cells expressing YFP-tagged AtPUB43 were infected by GFP-tagged oomycete P. capsici (artificially represented by the red color). Top panel: tile-scan imaging showing the changing distribution of YFP-AtPUB43 on the PM associated with the progress of P. capsici infection. Scale bar in this panel represents 50 μm. Lower panel: details of the distribution of YFP-AtPUB43 in necrotic (outlined by gray box), infected (outlined by purple box) and uninfected cells (outlined by blue box). (B) Distribution of ARA6-YFP compared to YFP-AtPUB43 in uninfected and infected cells. The tonoplast- and MVB-associated patches formed by YFP- AtPUB43 were revealed by ARA6-YFP as highlighted with open and filled arrows, respectively. Likewise, punctae associating with the tonoplast or MVBs, are highlighted by dotted and solid arrows, respectively. All other scale bars represent 10 μm. [file Image_12.JPEG]
